# Spermiogenesis and spermatozoon ultrastructure in basal polyopisthocotylean monogeneans, Hexabothriidae and Chimaericolidae, and their significance for the phylogeny of the Monogenea

**DOI:** 10.1051/parasite/2018007

**Published:** 2018-02-13

**Authors:** Jean-Lou Justine, Larisa G. Poddubnaya

**Affiliations:** 1 Institut Systématique Évolution Biodiversité (ISYEB), Muséum National d’Histoire Naturelle, CNRS, Sorbonne Université, EPHE, 57 rue Cuvier, CP 51, 75005 Paris France; 2 I. D. Papanin Institute for Biology of Inland Waters, Russian Academy of Sciences, 152742 Borok, Yaroslavl Russia

**Keywords:** Monogenea, phylogeny, sperm ultrastructure

## Abstract

Sperm ultrastructure provides morphological characters useful for understanding phylogeny; no study was available for two basal branches of the Polyopisthocotylea, the Chimaericolidea and Diclybothriidea. We describe here spermiogenesis and sperm in *Chimaericola leptogaster* (Chimaericolidae) and *Rajonchocotyle emarginata* (Hexabothriidae), and sperm in *Callorhynchocotyle callorhynchi* (Hexabothriidae). Spermiogenesis in *C. leptogaster* and *R. emarginata* shows the usual pattern of most Polyopisthocotylea with typical zones of differentiation and proximo-distal fusion of the flagella. In all three species, the structure of the spermatozoon is biflagellate, with two incorporated trepaxonematan 9 + “1” axonemes and a posterior nucleus. However, unexpected structures were also seen. An alleged synapomorphy of the Polyopisthocotylea is the presence of a continuous row of longitudinal microtubules in the nuclear region. The sperm of *C. leptogaster* has a posterior part with a single axoneme, and the part with the nucleus is devoid of the continuous row of microtubules. The spermatozoon of *R. emarginata* has an anterior region with membrane ornamentation, and posterior lateral microtubules are absent. The spermatozoon of *C. callorhynchi* has transverse sections with only dorsal and ventral microtubules, and its posterior part shows flat sections containing a single axoneme and the nucleus. These findings have important implications for phylogeny and for the definition of synapomorphies in the Neodermata. We point out a series of discrepancies between actual data and interpretation of character states in the matrix of a phylogeny of the Monogenea. Our main conclusion is that the synapomorphy “lateral microtubules in the principal region of the spermatozoon” does not define the Polyopisthocotylea but is restricted to the Mazocraeidea.

## Abstract (fr)

L’ultrastructure des spermatozoïdes fournit des caractères morphologiques utiles pour comprendre la phylogénie ; aucune étude n’était disponible pour deux branches basales des Polyopisthocotylea, les Chimaericolidea et des Diclybothriidea. Nous décrivons ici la spermiogenèse et le spermatozoïde chez *Chimaericola leptogaster* (Chimaericolidae) et *Rajonchocotyle emarginata* (Hexabothriidae), et le spermatozoïde chez *Callorhynchocotyle callorhynchi* (Hexabothriidae). La spermiogenèse chez *C. leptogaster* et *R. emarginata* montre le schéma habituel de la plupart des Polyopisthocotylea avec une zone de différenciation typique et une fusion proximo-distale des flagelles. Chez les trois espèces, la structure du spermatozoïde est biflagellée, avec deux axonèmes incorporés de type Trepaxonemata 9 + “1” et un noyau postérieur. Cependant, des structures inattendues ont également été observées. Une synapomorphie présumée des Polyopisthocotylea est la présence d’une rangée continue de microtubules longitudinaux dans la région nucléaire. Le spermatozoïde de *C. leptogaster* a une partie postérieure avec un seul axonème, et la partie avec le noyau est dépourvue de la rangée continue de microtubules. Le spermatozoïde de *R. emarginata* a une région antérieure avec ornementation membranaire et les microtubules postérieurs latéraux sont absents. Le spermatozoïde de *C. callorhynchi* a des coupes transversales montrant seulement des microtubules dorsaux et ventraux, et sa partie postérieure présente des coupes plates contenant un seul axonème et le noyau. Ces observations ont des implications importantes pour la phylogénie et la définition des synapomorphies des Neodermata. Nous signalons une série de divergences entre les données réelles et l’interprétation des états de caractères dans la matrice d’une phylogénie des Monogenea. Notre conclusion principale est que la synapomorphie « microtubules latéraux dans la région principale du spermatozoïde » ne définit pas les Polyopisthocotylea mais est limitée aux Mazocraeidea.

## Introduction

Sperm ultrastructure has been used as a source of significant characters to elucidate phylogenetic relationships in the Platyhelminthes [20,21], and especially in the parasitic Platyhelminthes or Neodermata. This was the case for the Neodermata as a whole [35,39,42], the Cestoda [2,29,41,67], the Digenea ([6,39] and many recent references, *e.g.* [5,7,8,73]) and the Monogenea [12–14,22,34,39,46].

In the Monogenea, the Monopisthocotylea and the Polyopisthocotylea have each been considered to bear respective sperm synapomorphies [34], but no spermatological character has been found to unite the two groups [40].

The Monopisthocotylea have revealed important variations of sperm structure, which led to the recognition of several synapomorphies [34,39,42] that were used in combination with other morphological characters to build phylogenies [12,14]. In contrast, the Polyopisthocotylea show a relatively uniform sperm morphology [34,39] with the significant exception of the Diplozoidae with an aberrant aflagellate spermatozoon [47]; the latter has been considered to be related to the exceptional biology of fertilization in diplozoids and especially the absence of sperm competition [39,47].

The Polyopisthocotylea include, in modern classifications [26], four orders: the Polystomatidea, Chimaericolidea, Diclybothriidea and Mazocraeidea. The spermatozoon and sometimes spermiogenesis ultrastructure are documented in several species of the Polystomatidae, in one species of Sphyranuridae (Polystomatidea), and in many families belonging to the Mazocraeidea (Table 1). However, no information was available for the Chimaericolidea and Diclybothriidea. Since the Chimaericolidea or these two orders were considered basal to the Mazocraeidea in both morphological [12] and molecular [32,75,78] analyses, missing data on sperm structure in these orders was a significant knowledge gap of sperm structure in Polyopisthocotylea, and even of the Neodermata as a whole.

**Table 1:** Studies of sperm ultrastructure in the Polyopisthocotylea.

Family (Alpha. order)	Genus, species, author, and host *	Sperm reference	“Polyopisthocotylean” structure of sperm **	Details **
Axinidae	*Axine* sp. (ex *Hemirhamphus brasiliensis*)	[55]	no	Only two micrographs; no lateral microtubules
Chauhaneidae	*Pseudomazocraes* cf. *monsivaisae*	[55]	Polyopisthocotylean	Typical structure; extremity with nucleus + single axoneme
Diclidophoridae	*Diclidophora merlangi* (Kuhn in Nordmann, 1832) Krøyer, 1851	[27]	Polyopisthocotylean	Includes spermiogenesis; single micrograph shows typical structure
Diclidophoridae	*Diclidophora merlangi* (Kuhn in Nordmann, 1832) Krøyer, 1851	[70]	Polyopisthocotylean	Single micrograph shows typical structure
Diclidophoridae	*Diclidophora* sp.	[105]	Polyopisthocotylean	Only two micrographs, but typical sections of spermatozoa
Diclidophoridae	*Choricotyle chrysophryi* Van Beneden & Hesse, 1863 [as *Choricotyle pagelli*]	[105]	Polyopisthocotylean	A single micrograph
Diplozoidae	*Diplozoon* sp.? [as *Diplozoon gracile*]	[39,48]	Aberrant	No axonemes; numerous parallel microtubules
Discocotylidae	*Discocotyle sagittata* (Leuckart, 1842)	[19]	Polyopisthocotylean	Includes spermiogenesis; crescent-shaped nucleus; lateral flange
Gastrocotylidae	*Pricea multae* Chauhan, 1945	[110]	Polyopisthocotylean	Includes spermiogenesis; typical section; lateral flange
Gastrocotylidae	*Gastrocotyle* sp.	[99]	Polyopisthocotylean	Includes spermiogenesis; no section with nucleus and both axonemes, but sections with nucleus and single axoneme have the lateral microtubules
Gotocotylidae	*Gotocotyla acanthura* (Parona & Perugia, 1896) Meserve, 1938 (as *Gotocotyla secunda*)	[92]	dubious	Single photograph, without nucleus section
Gotocotylidae	*Gotocotyla acanthura* (Parona & Perugia, 1896) Meserve, 1938	[56]	Polyopisthocotylean	Typical polyopisthocotylean structure with additional undulating membrane
Heteraxinidae	*Cemocotyle* sp. (ex *Caranx crysos*)	[58]	Polyopisthocotylean	Single micrograph, but typical
Heteraxinidae	*Heteraxine* sp. (ex *Scomberomorus tritor*)	[58]	Polyopisthocotylean	Several micrographs, typical
Heteraxinidae	*Heteraxinoides* sp. (ex *Pomadasys jubelini*)	[58]	dubious	Single photograph, not in region of nucleus
Heteraxinidae	*Gonoplasius* sp. (ex *Pseudocaranx dentex*)	[94]	Polyopisthocotylean	Includes spermiogenesis
Hexabothriidae	*Erpocotyle catenulata* (Guberlet, 1933)	[105]	Polyopisthocotylean	Single micrograph with several sections; electron-lucent nucleus; membranes disrupted; not sure whether lateral microtubules are present
Hexostomatidae	*Hexostoma* sp. (ex *Euthynnus alleteratus*)	[54]	Polyopisthocotylean	Only three sections shown, but typical
Mazocraeidae	*Kuhnia* sp. (ex *Scomber colias* [as *S. japonicus*])	[55]	Polyopisthocotylean	Only two micrographs; typical structure, additional cytoplasmic microtubules
Microcotylidae	*Metamicrocotyla macracantha* (Alexander, 1954) Koratha, 1955	[9]	Polyopisthocotylean	Includes spermiogenesis
Microcotylidae	*Microcotyle* sp.	[10]	Polyopisthocotylean	Includes spermiogenesis; only two micrographs depict typical sperm structure
Microcotylidae	*Polylabroides australis* (Murray, 1931) Mamaev & Parukhin, 1976	[95]	Polyopisthocotylean	Includes spermiogenesis
Microcotylidae	*Pagellicotyle mormyri* (Lorenz, 1878) Mamaev, 1984 [as *Microcotyle mormyri*]	[104,105]	Polyopisthocotylean	Includes spermiogenesis
Microcotylidae	*Microcotyle erythrini* Van Beneden & Hesse, 1863	[105]	Polyopisthocotylean	Includes spermiogenesis
Microcotylidae	*Atrispinum sargi* (Parona & Perugia, 1890) Euzet & Maillard, 1974 [as At. “*sargui*”]	[33,34]	Polyopisthocotylean	Not illustrated
Microcotylidae	*Atriaster* sp. (ex *Diplodus cervinus*)	[37]	Polyopisthocotylean	Includes spermiogenesis; polygonal nucleus
Microcotylidae	*Atriaster heterodus* Lebedev & Parukhin, 1969	[96]	Polyopisthocotylean	Includes spermiogenesis; polygonal nucleus
Microcotylidae	*Microcotyle* sp. (ex *Abudefduf analogus*)	[55]	Polyopisthocotylean	Typical structure; membrane ornamentation
Microcotylidae	*Sciaenacotyle panceri* (Sonsino, 1891) Mamaev, 1989 [as *Microcotyle pancerii*]	[89]	Polyopisthocotylean	Includes spermiogenesis
Octomacridae	*Octomacrum lanceatum* Mueller, 1934	[28]	Polyopisthocotylean	Includes spermiogenesis; typical sections
Plectanocotylidae	*Plectanocotyle gurnardi* (Van Beneden & Hesse, 1863)	[105]	Polyopisthocotylean	Only two micrographs, but typical structure well visible
Polystomatidae	*Neopolystoma spratti* Pichelin, 1995	[109]	no	Includes spermiogenesis; at level of nucleus, the longitudinal microtubules do not form a complete circle
Polystomatidae	*Pseudodiplorchis americanus* Rodgers & Kuntz, 1940) Yamaguti, 1963	[18]	no	Includes spermiogenesis; sections with nucleus have no lateral microtubules
Polystomatidae	*Polystoma* sp.	[68]	no	Sections with nucleus have no lateral microtubules
Polystomatidae	*Polystoma* sp.	[11]	dubious	Not illustrated; reports absence of peripheral microtubules
Polystomatidae	*Concinnocotyla australensis* (Reichenbach- Klinke, 1966) Pichelin, 1991	[110]	Polyopisthocotylean	Includes spermiogenesis; annular nucleus section wrapping axonemes; two lateral flanges
Polystomatidae	*Polystomoides* sp.	[90,91]	dubious	No section at the level of nucleus
Protomicrocotylidae	*Protomicrocotyle ivoriensis* Wahl, 1972	[99]	Polyopisthocotylean	Includes spermiogenesis
Pterinotrematidae	*Pterinotrema* sp.	[34]	no	Not illustrated; the text states that lateral microtubules are absent
Pyragraphoridae	*Pyragraphorus pyragraphorus* (MacCallum & MacCallum, 1913) Sproston, 1946	[55]	Polyopisthocotylean	Two micrographs; typical structure
Sphyranuridae	*Sphyranura* sp.	[34]	dubious	Not illustrated; based on “personal communication by Oliver and Euzet”

* The systematic placement of species has been updated when appropriate; the host fish is indicated when the monogenean species was not identified.

** “Polyopisthocotylean”: The Polyopisthocotylean structure is defined as two axonemes and a continuous row of peripheral microtubules in the region which contains the nucleus; “Aberrant”: special case of Diplozoidae; “Dubious”: available micrographs do not show the Polyopisthocotylean structure; “no”: available micrographs show that the structure is not polyopisthocotylean.

In this paper we present, for the first time, observations on two species of the family Hexabothriidae (Diclybothriidea) and one of the family Chimaericolidae (Chimaericolidea), thus filling the gaps in our knowledge of sperm ultrastructure in the Monogenea. These observations complement previous studies on the tegument [88], attachment organs [84,85], reproductive organs [83,87], and digestive system [86] of the same species.

## Material and Methods

For electron microscopy, adult specimens of three polyopisthocotylean monogeneans were recovered from the gills of naturally infected cartilaginous fishes: *Chimaericola leptogaster* (Leuckart, 1830) (Chimaericolidae) from the chimaera (rabbit fish) *Chimaera monstrosa* Linnaeus, 1758 (Holocephali), *Rajonchocotyle emarginata* (Olsson, 1876) (Hexabothriidae) from the thorny (starry) ray *Amblyraja radiata* (Donovan, 1808) (Elasmobranchii), and *Callorhynchocotyle callorhynchi* (Manter, 1955) (Hexabothriidae) from the chimaera (Cape elephant fish) *Callorhynchus capensis* Duméril, 1865 (Holocephali). The first two were collected in the Norwegian Sea off Tromsø, Norway, and the latter was from the Southeast Atlantic off St Helena Bay on the western coast of South Africa. Live specimens of all three monogenean species were fixed using 3 % glutaraldehyde in 0.1 M sodium cacodylate buffer (pH 7.2) for 20 days at 5 °C, rinsed four times for 20 min in the same buffer and post-fixed in 1 % osmium tetroxide for 1 h. For ultrathin studies, samples were then dehydrated in a graded series of ethanol and acetone, and embedded in a mixture of Araldite and Epon. Ultrathin sections (70–90 nm in thickness) were stained with uranyl acetate and lead citrate, and examined using a JEOL-JEM-1011 transmission electron microscope operating at 80 kV [86].

Observations with the electron microscope were performed by LGP; interpretation and choice of micrographs to be included in this study were done by JLJ.

All transverse sections of spermatozoa and spermatids in the figures, with a few exceptions when several sections are together in a micrograph, are orientated with the mitochondrion at the bottom, thus following the arbitrary convention of Sato, Oh & Sakoda [97] of mitochondrion as “ventral”. Longitudinal sections of spermatids are orientated with the anterior part at the top, *i.e.*, for the zone of differentiation, the arched membranes at the top, and with the free flagella and the median cytoplasmic process at the bottom [35,39]. Longitudinal schematic drawings of spermatozoa are oriented with the centrioles at the top and the nucleus at the bottom, since neodermatan spermatozoa are “inverted” in comparison with most phyla [1,39].

## Results

### Spermiogenesis in the chimaericolid *Chimaericola leptogaster* (Figures 1 and 2)

**Figure 1:**
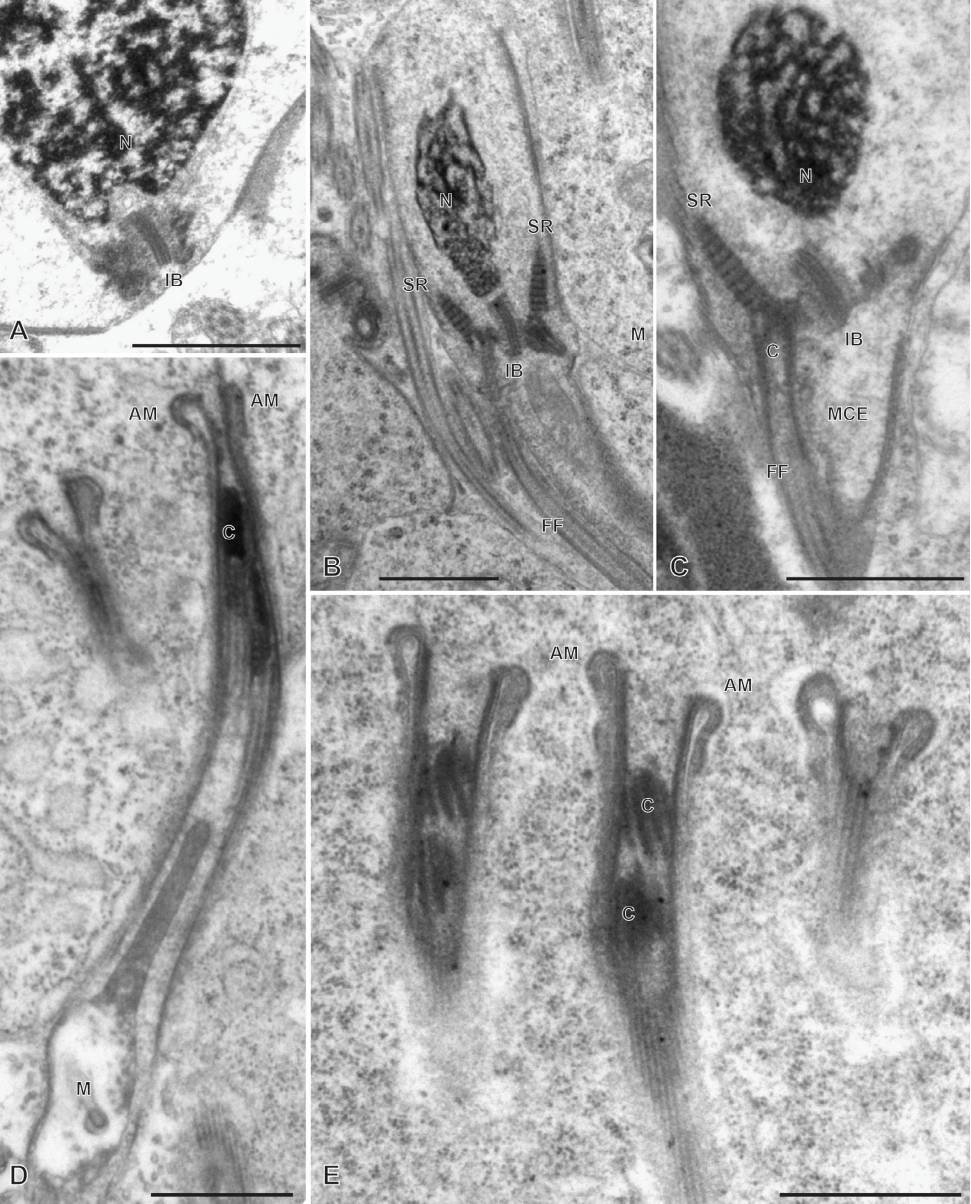
Early spermiogenesis in *Chimaericola leptogaster*. A, early zone of differentiation. Intercentriolar body next to cell membrane. B, C, fully developed zone of differentiation, longitudinal sections. Intercentriolar body flanked by two centrioles, each associated with a striated root. Two free flagella and one median cytoplasmic process are attached at the distal extremity of the zone of differentiation. D, E, longitudinal sections of late zones of differentiation, embedded in the cytoplasm of the mass of spermatids. Arching membranes at the proximal extremity of each zone of differentiation. Scale in all figures: 1 μm.

**Figure 2:**
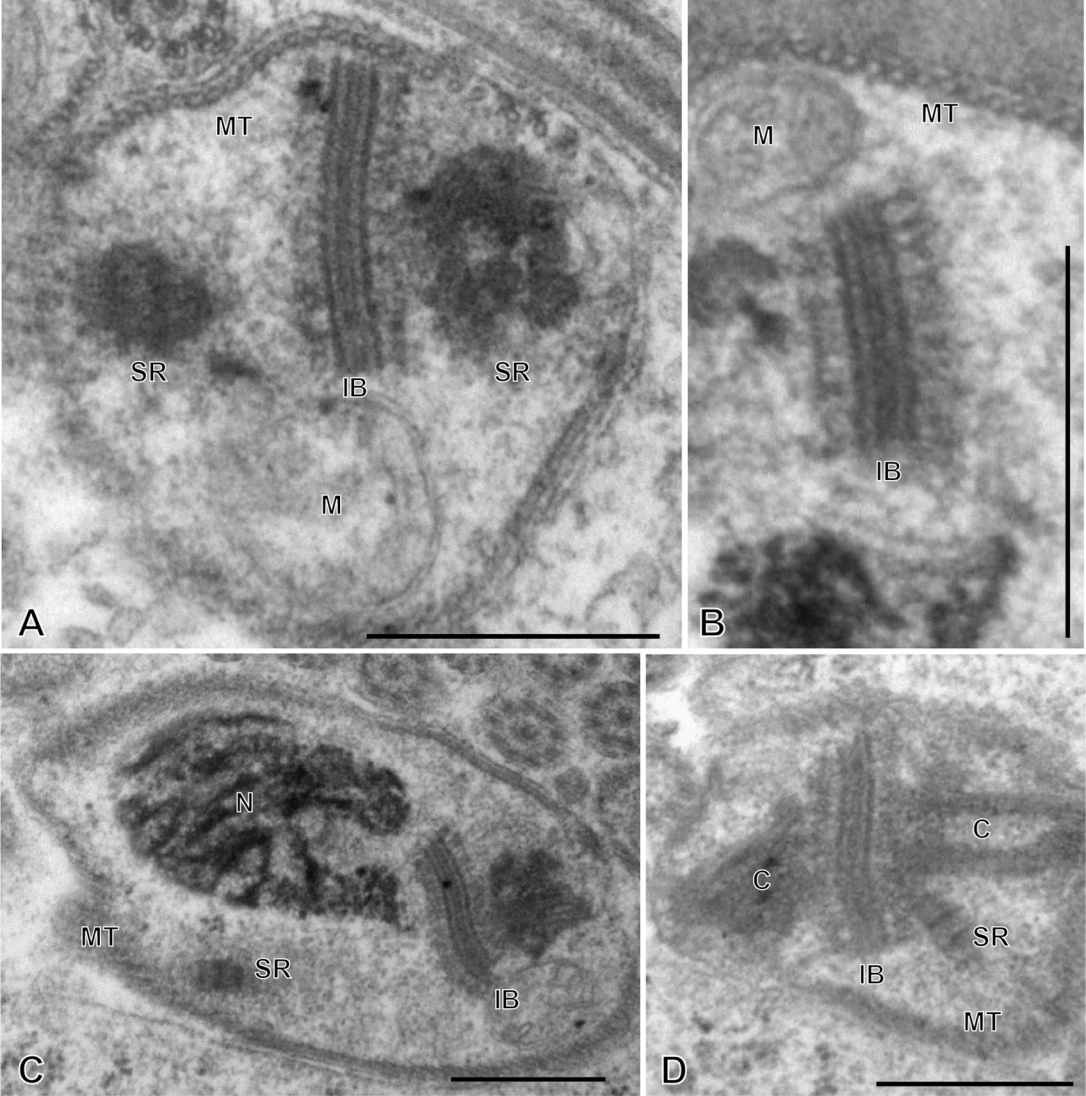
Spermiogenesis in *Chimaericola leptogaster* (continued). A, transverse section of fully developed zone of differentiation, at the level of the two striated roots and the intercentriolar body. B, detail of intercentriolar body and peripheral microtubules. C, D, various oblique sections of intercentriolar body, centriole and striated roots. Scale in all figures: 500 nm.

Spermiogenesis involves the formation of a protuberance on each spermatid, termed zone of differentiation. The early zone of differentiation is visible as a small protuberance which is close to the extremity of the nucleus. An intercentriolar body is perpendicular to the cell membrane; the membrane shows peripheral microtubules (Figure 1A). The mature zone of differentiation has the typical structure found in most neodermatans (Polyopisthocotylea, Digenea and Cestoda). It is conical, with arching membranes at its base (proximal extremity) and bears, at its distal extremity, two lateral free flagella and a median cytoplasmic process. The zone of differentiation contains two centrioles flanking the intercentriolar body and a longitudinal striated root is associated with each centriole (Figures 1B, 1C, 2). The mitochondrion and the nucleus pass through the zone of differentiation (Figures 1B, 1C, 2) into the median cytoplasmic process (Figure 1B). The two flagella fuse with the median cytoplasmic process; the late zone of differentiation contains the two centrioles, slightly slanted (Figure 1E) and has arching membranes at its proximal extremity (Figure 1D, 1E).

### Spermatozoon of the chimaericolid *Chimaericola leptogaster* (Figures 3 and 4)

**Figure 3:**
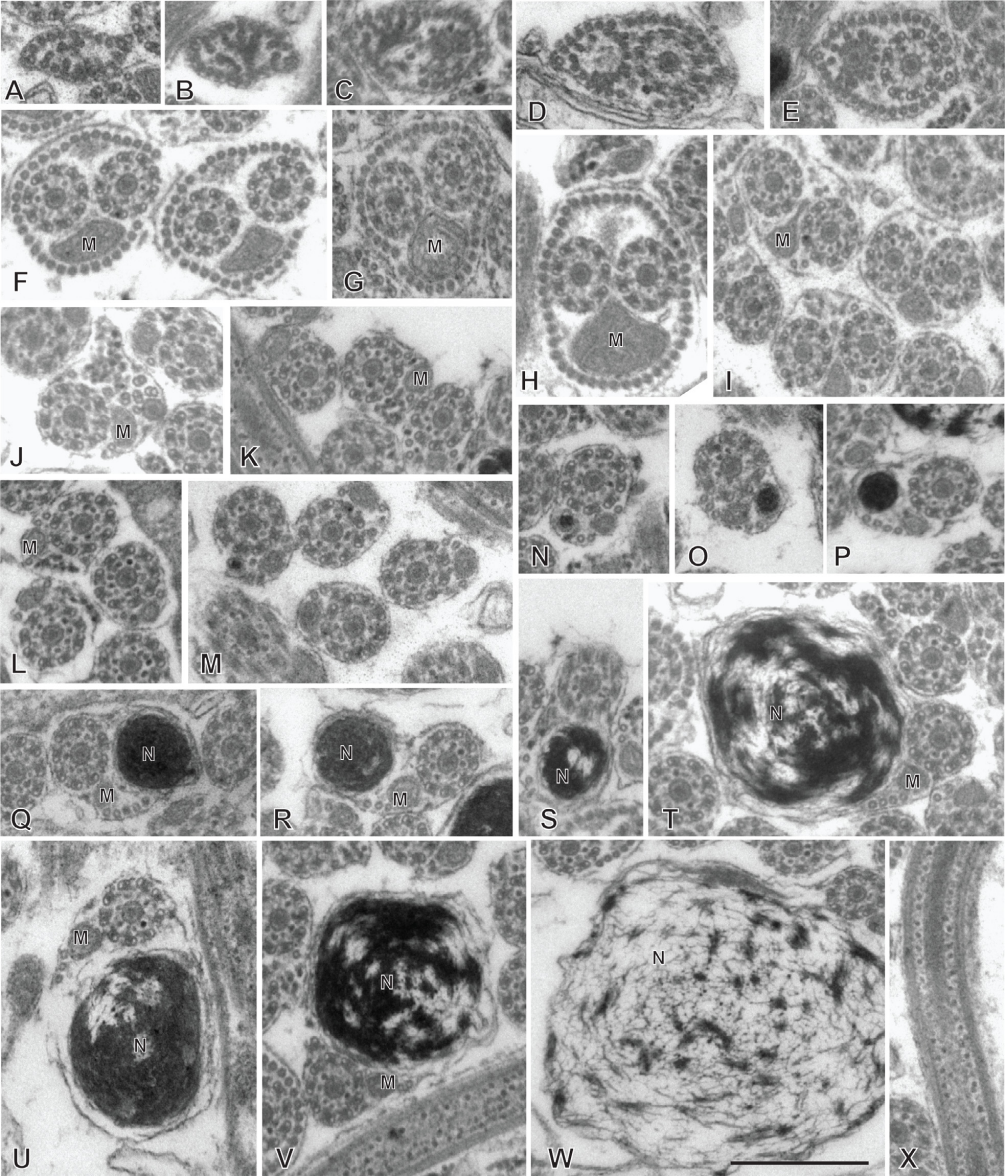
Spermatozoon of *Chimaericola leptogaster*. A-W, transverse sections, in antero-posterior sequence; X, longitudinal section. A-C, anteriormost part of spermatozoon; peripheral microtubules and progressive appearance of the two centrioles. D, E, one fully formed axoneme and section of the centriole of the other axoneme. F-H, peripheral microtubules (full ring in G,H), two axonemes, and mitochondrion. I, two axonemes, peripheral row of microtubules reduced to a few units, and mitochondrion. J, one axoneme, distal extremity of other axoneme as scattered doublets and central core, a few remaining peripheral microtubules, and mitochondrion. K-M, one axoneme and mitochondrion, a few peripheral microtubules. N-P, one axoneme, a few peripheral microtubules, mitochondrion and section of the anterior thin part of the nucleus, which is wider as sections are more posterior. Q-S, one axoneme, a few peripheral microtubules, mitochondrion, and section of nucleus approximately as wide as axoneme. T-W, one axoneme, a few peripheral microtubules, mitochondrion, and wide section of nucleus. W is a very wide section with electron-transparent chromatin. X, typical trepaxonematan axoneme. Scale in W, valid for all figures: 500 nm.

The mature spermatozoon is a very elongate and thin cell; the anteroposterior sequence of transverse sections was deduced from comparison of sections, based on the simple principle that the nucleus is posterior and the centrioles are anterior, as in all neodermatans [35,39]. The axonemes show a typical trepaxonematan 9 + “1” structure (Figure 3E-W, transverse sections; Figure 3X, longitudinal section).

**Figure 4:**
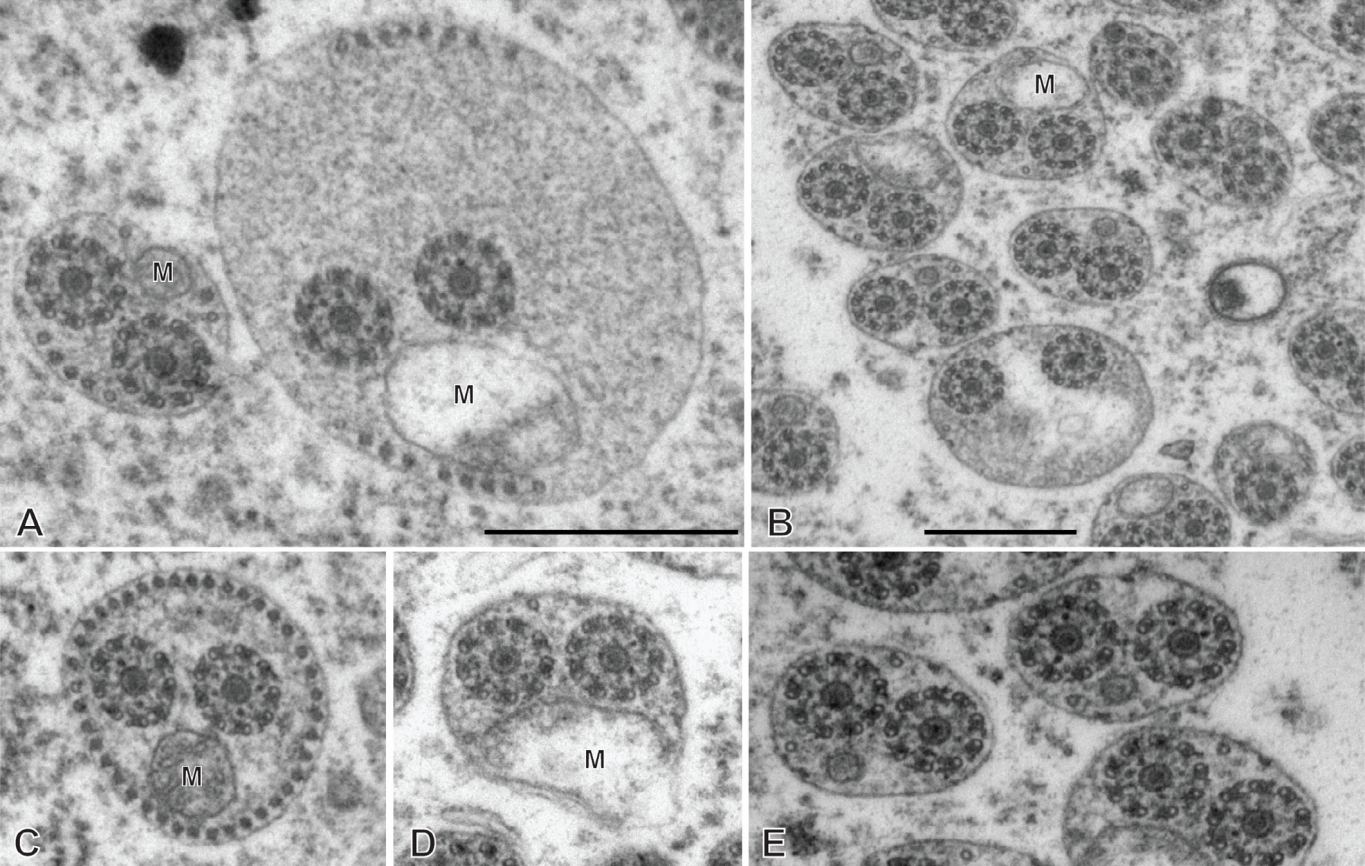
Altered spermatozoa of *Chimaericola leptogaster* in caeca and genito-intestinal canal. A, B, transverse sections of altered spermatozoa in caecum, showing swollen cytoplasm and swollen mitochondrion without crests. C, a typical unaltered spermatozoon section in caecum, probably corresponding to a spermatozoon just transferred to this organ. D, E, transverse sections of altered spermatozoa in genito-intestinal canal. Scale in A, valid for A, C, D, E. All scales: 500 nm.

The general structure of the spermatozoon is biflagellate, with two axonemes incorporated into the sperm body, a longitudinal mitochondrion, a nucleus, and cortical longitudinal microtubules. The anterior region of the spermatozoon is pointed; it shows a few peripheral microtubules and the centriolar derivative of one of the axonemes (Figure 3A-B). More posteriorly, sections show the second centriolar derivative (Figure 3C) and the first axoneme, surrounded by a continuous row of peripheral microtubules (Figure 3D-E). The next region, in an antero-posterior sequence, contains the two axonemes, the peripheral row of microtubules and a section of the mitochondrion (Figure 3F-H). The following region has only a few peripheral microtubules (Figure 3I-M); one of the axonemes finishes (as doublets, Figure 3J) and thus the rest of the cell contains a single axoneme. The anteriormost sections in this region show only the mitochondrion, which has a small diameter in cross sections, but more posterior sections show the first sections of the nucleus which are sections with very small diameter (Figure 3N-P). The posterior region of the spermatozoon contains a single axoneme, a few peripheral microtubules, a small diameter section of the mitochondrion, and a section of the nucleus with a diameter increasing towards the posterior end (Figure 3Q-W). The chromatin of the nucleus is compact and electron-dense in its thin, anterior part (Figure 3N-Q) then partly compact and partly fibrous (Figure 3R-V), and finally fibrous and electron-lucent in its posteriormost part (Figure 3W). The distal posterior extremity of the spermatozoon contains only the nucleus and a single axoneme (Figure 3W).

**Figure 13:**
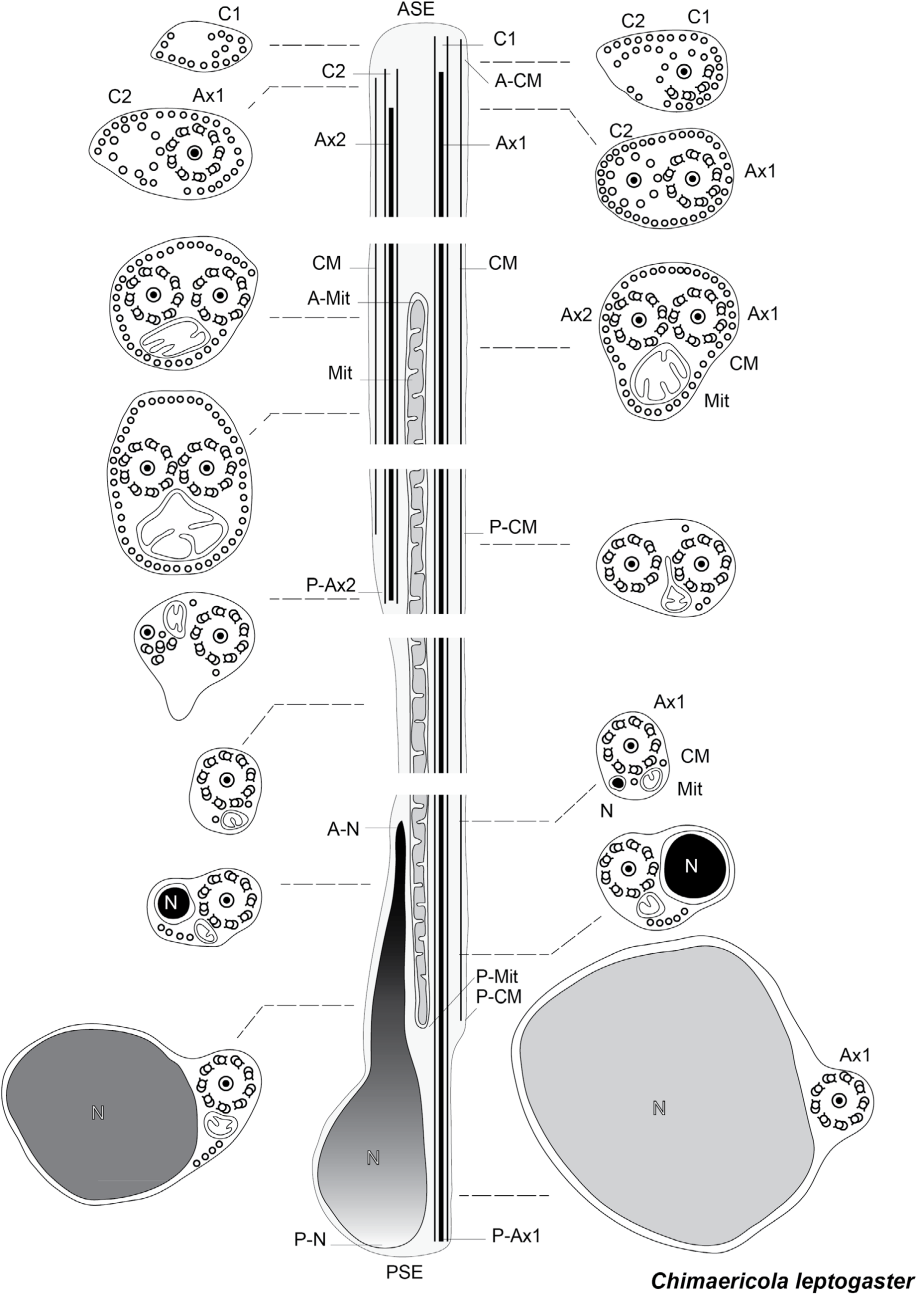
Schematic reconstruction of the spermatozoon of *Chimaericola leptogaster.*

Figure 13 is a schematic drawing of our interpretation of the structure of the mature spermatozoon of *Chimaericola leptogaster*.

While useful information about sperm structure should be sought only from sections of spermatozoa in the reproductive organs, we had the opportunity to observe sections in the caeca and in the genito-intestinal canal. Sections were generally indicative of cells in hypotonic media, with swollen cytoplasm with uniform contents and swollen mitochondria (Figure 4A, B, D, E). Occasionally, a few sections which seemed normal (Figure 4C, compare with Figure 3G) were found, and probably correspond to spermatozoa which were recently transferred to these organs.

### Spermiogenesis in the hexabothriid *Rajonchocotyle emarginata* (Figures 5-7)

**Figure 5:**
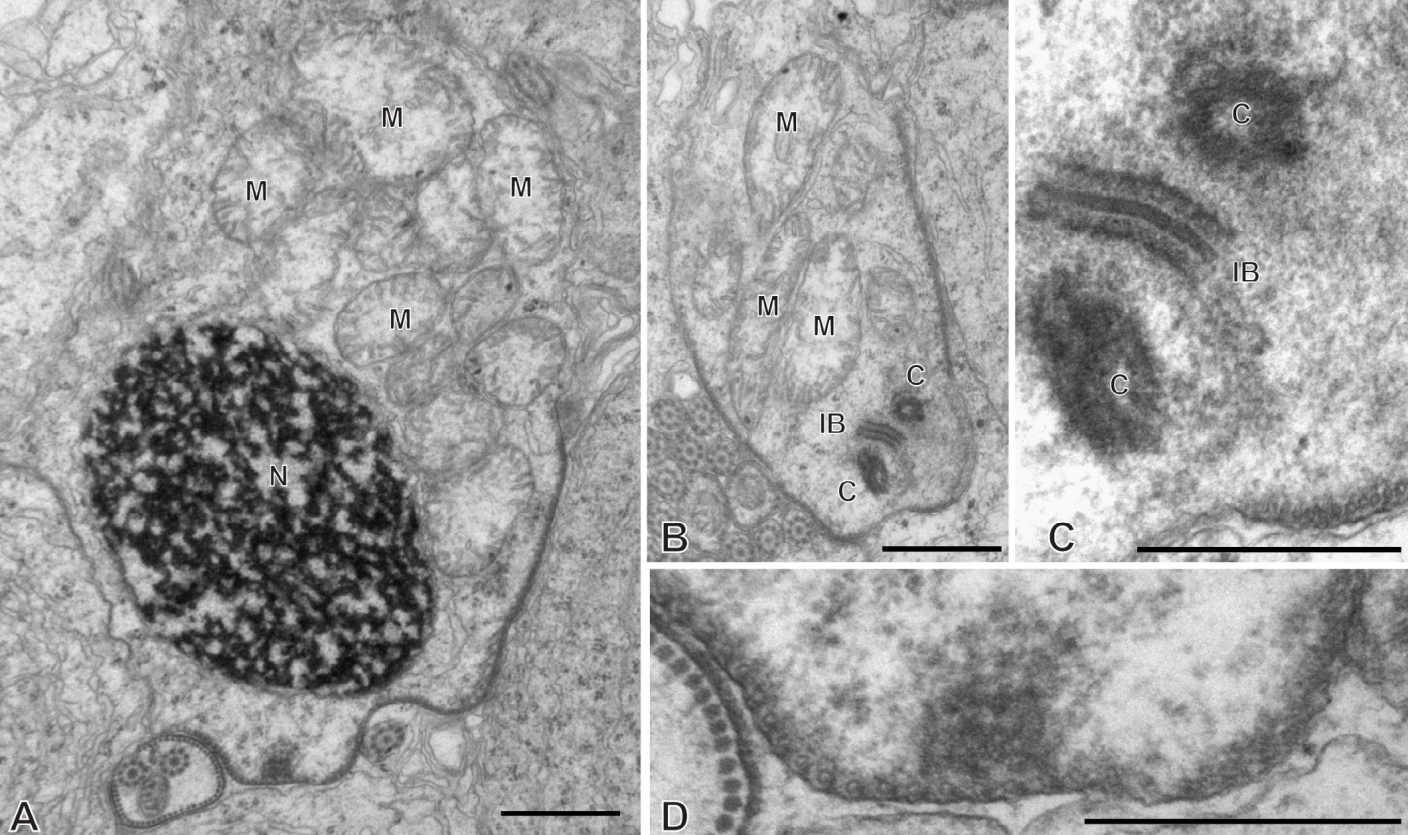
Early spermiogenesis in *Rajonchocotyle emarginata*. A, B, very early spermatid with round nucleus and mitochondria gathered above nucleus. The early zone of differentiation is lined with microtubules; constituents of the zone of differentiation visible as a densification of the cytoplasm. C, early zone of differentiation, with fully formed intercentriolar body and two centrioles. This section does not cut the nucleus. D, magnification of early zone of differentiation in A. Scales in all figures: 500 nm.

**Figure 6:**
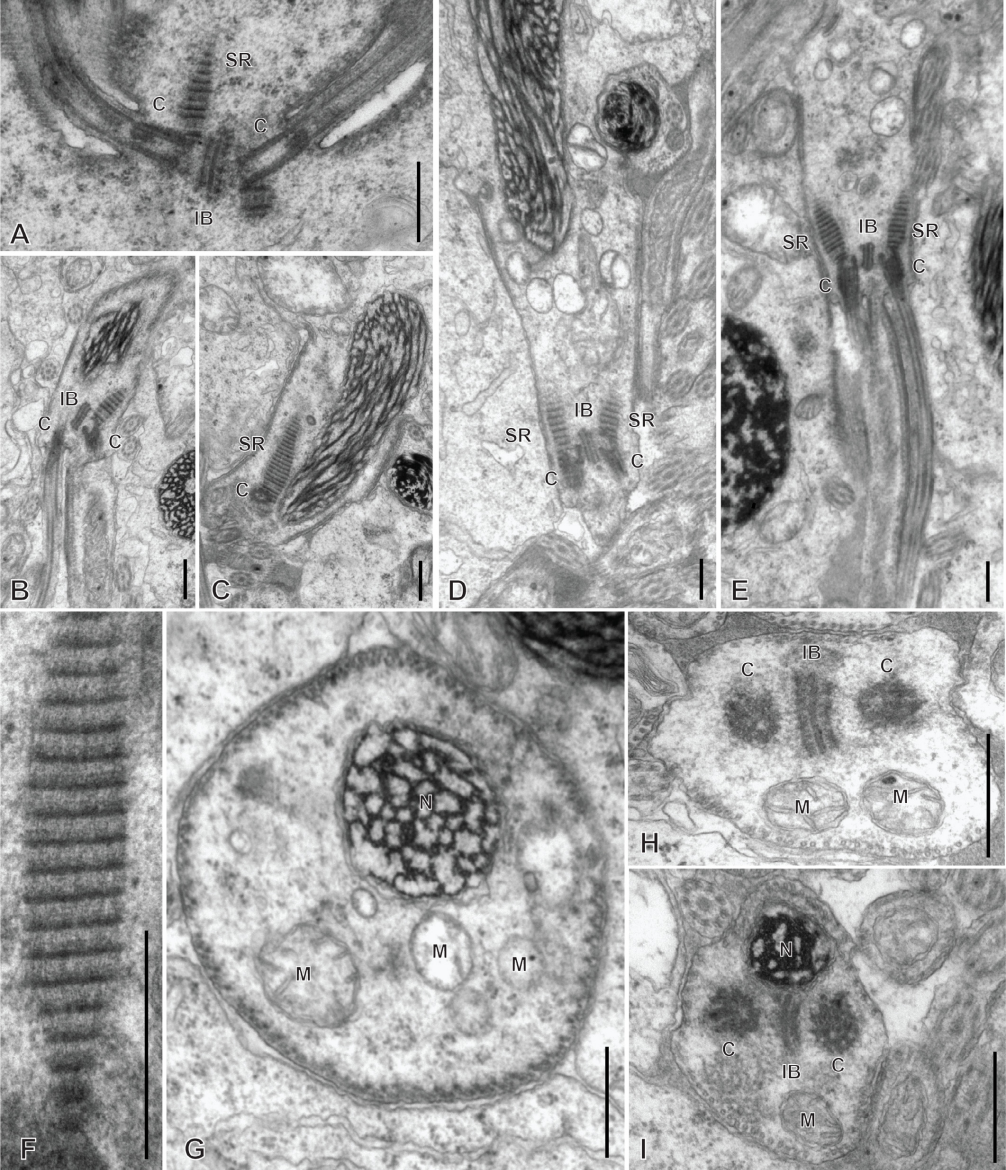
Spermiogenesis in *Rajonchocotyle emarginata*, fully formed zones of differentiation. A, zone of differentiation at stage when free flagella are directed backwards along the zone of differentiation. B-E, longitudinal sections of fully formed zones of differentiation, showing typical morphology with elongate nucleus with lamellated chromatin, two centrioles, two striated roots, two free flagella and median cytoplasmic process. Mitochondria appear separate and roundish. F, high magnification of striated root, longitudinal section. G-H, transverse sections of fully formed zones of differentiation; row of peripheral microtubules in all sections. G, section in proximal part of zone of differentiation, showing nucleus and several mitochondria. H, I, sections at level of intercentriolar body and centrioles, with nucleus and/or mitochondria. Scales in all figures: 500 nm.

**Figure 7:**
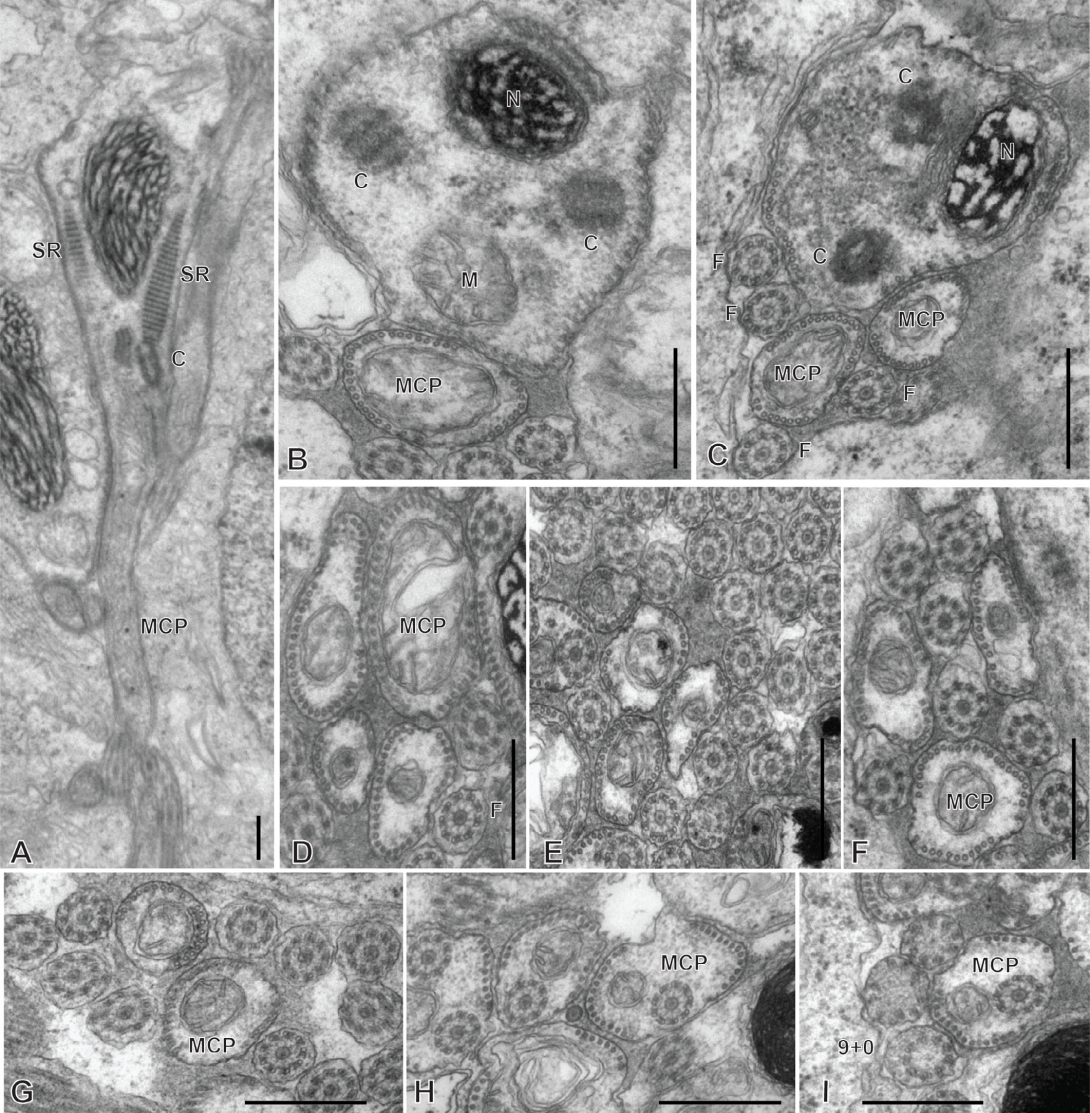
Spermiogenesis in *Rajonchocotyle emarginata*, fully formed zones of differentiation and median cytoplasmic process. A, longitudinal section of fully formed zone of differentiation showing long median cytoplasmic process at its distal extremity. B, C, side-by-side sections in zone of differentiation, with nucleus and/or mitochondrion, and median cytoplasmic process, with section of elongating mitochondrion; free flagella, with 9 + “1” trepaxonematan structure, are located along the median cytoplasmic process and parallel to it. D-G, sections of median cytoplasmic process with various numbers of peripheral microtubules. H, median cytoplasmic process with only one axoneme incorporated. I, transverse section of distal extremity of free flagella showing 9 + 0 structure. Scales in all figures: 500 nm.

**Figure 8:**
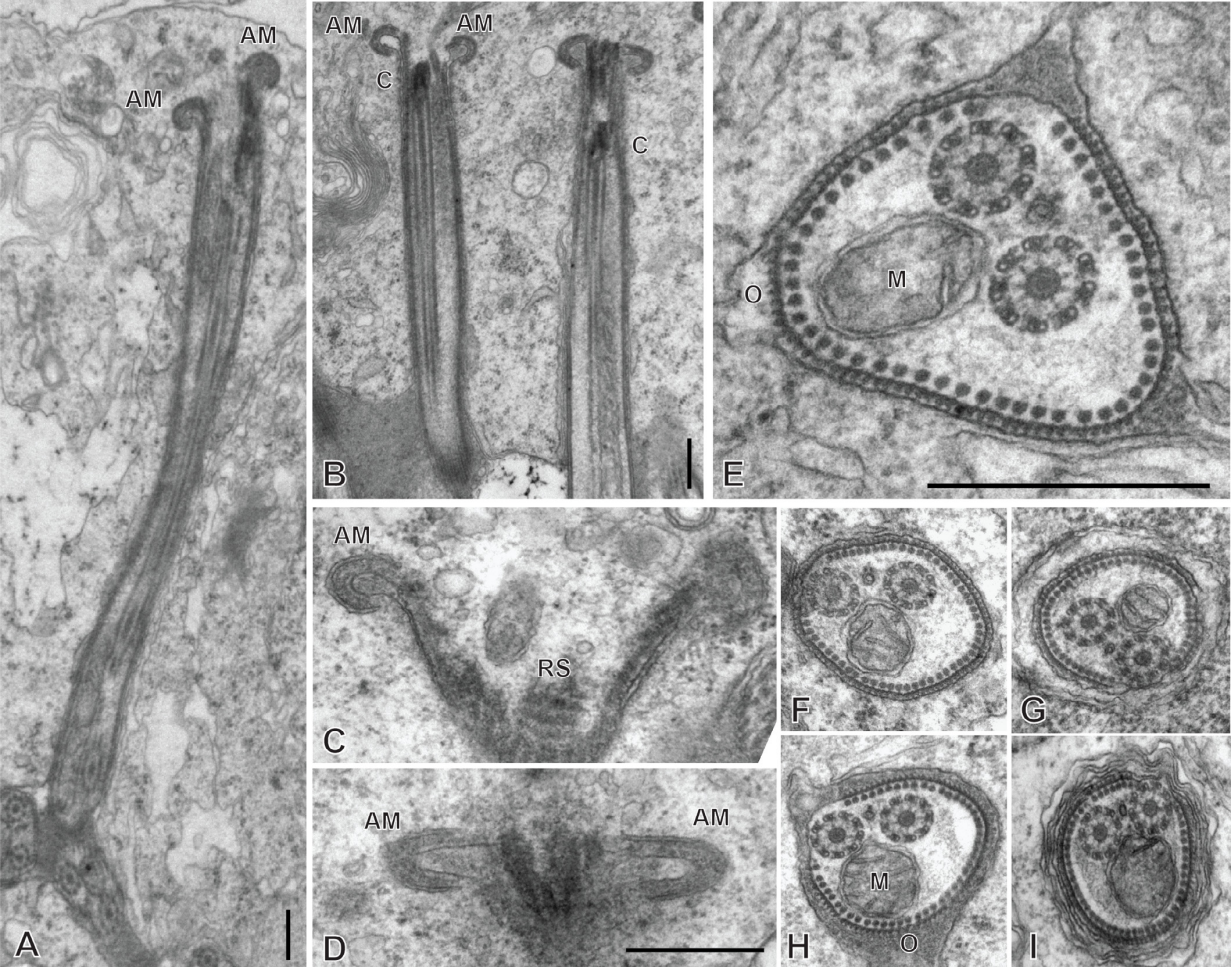
Spermiogenesis in *Rajonchocotyle emarginata*, late zones of differentiation. A, B, longitudinal sections of late zones of differentiation embedded in the cytoplasm of the mass of spermatids. Arching membranes at the anterior (proximal) extremity. C, D, high magnification of arching membranes. E, high magnification of transverse section of late zone of differentiation. Two fully-incorporated axonemes, one section of mitochondrion, and almost complete row of peripheral longitudinal microtubules. Note dense material external to cell membrane, or “ornamentation”. F-I, various transverse sections of late zones of differentiation; the cytoplasm of the surrounding spermatid is often lined with membranes (several layers in I). Scale in D valid for C- I. Scales in all figures: 500 nm.

Spermiogenesis involves the formation of a zone of differentiation. The early zone of differentiation is visible as a small protuberance with subpellicular microtubules (Figure 5A, D); at this stage, the nucleus is round and the mitochondria are gathered above the nucleus. Slightly more advanced zones of differentiation show the formation of the intercentriolar body and two centrioles, close to the cell membrane (Figure 5C, D). The two centrioles give rise to two free flagella which are first in an inversed position, with their distal extremities directed toward the basal part of the spermatid (Figure 6A). The next stage is a typical, conical, zone of differentiation, which contains peripheral microtubules, the elongating nucleus with lamellated chromatin, roundish mitochondria, and two centrioles each associated with a striated root and located on each side of the intercentriolar body. The distal extremity of the zone of differentiation bears three almost parallel processes, the two lateral free flagella, and the median cytoplasmic process (Figure 6B-I). The mitochondria seem to be separate, as roundish discrete elements, in the zone of differentiation (Figure 6D, E, G-I) but are fused into a single ribbon in the median cytoplasmic process (Figure 7). The median cytoplasmic process contains the mitochondrial ribbon and two sets of peripheral microtubules, one ventral and one dorsal (Figure 7B-G). Electron-dense zones of the membrane, known as “attachment zones” are visible on each side of the median cytoplasmic process (two on each side), where no peripheral microtubules are present (Figure 7B). The free flagella show the typical trepaxonematan 9 + “1” structure (Figure 7B-H) but their distal extremities lack the central core, thus producing distal sections with 9 + 0 structure (Figure 7I). The free flagella fuse with the median cytoplasmic process on its lateral sides; one flagellum fuses before the other, thus producing sections with a single axoneme incorporated into the median cytoplasmic process (Figure 7H). The advanced zone of differentiation is elongate and deeply embedded in a canal into the cytoplasm of the spermatid mass (Figure 8). Arching membranes are visible at the anterior (proximal) part (Figure 8A-D), and the transverse section of the canal shows that it is often surrounded by additional membranes (Figure 8G, 8I). At this stage, transverse sections of the zone of differentiation show a very characteristic structure, with two axonemes, a section of mitochondrion and an almost complete peripheral row of parallel longitudinal microtubules (Figure 8E-I). A particularity at this stage is the presence of ornamentation external to the membrane along the periphery, except where microtubules are absent (Figure 8E-I).

### Spermatozoon of the hexabothriid *Rajonchocotyle emarginata* (Figures 9 and 10)

**Figure 9:**
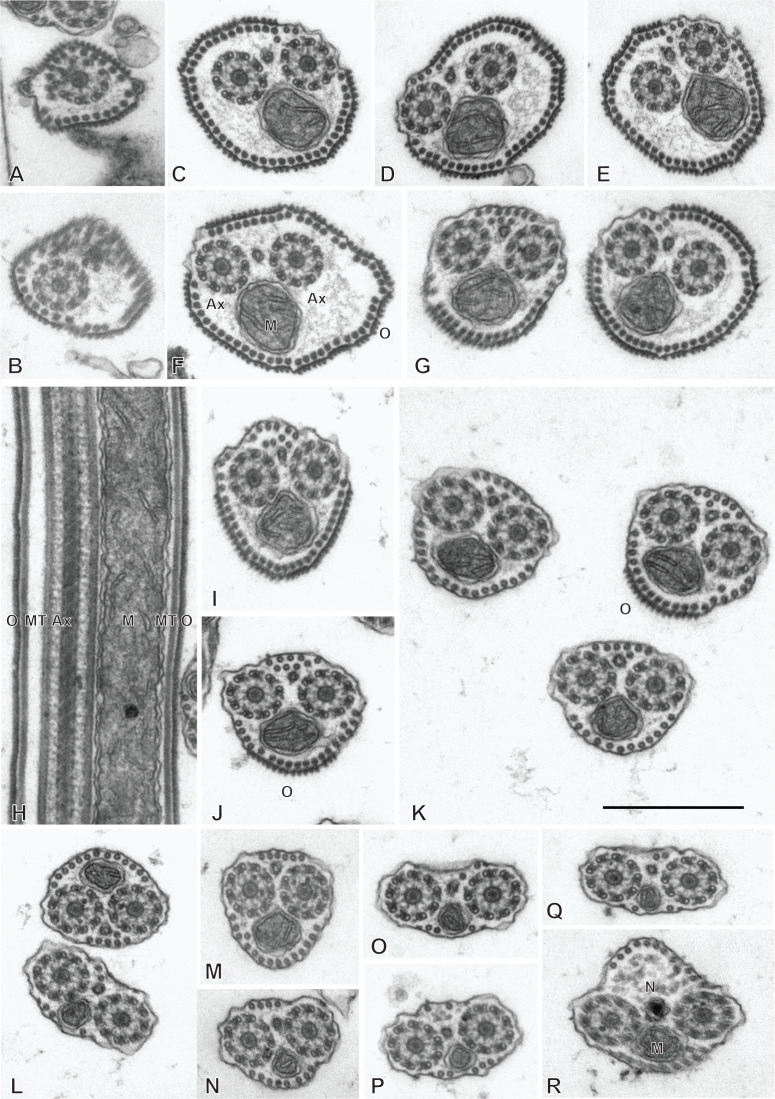
Spermatozoon of *Rajonchocotyle emarginata*, anterior part. A-G and I-R, transverse sections, in antero-posterior sequence; H, longitudinal section. A, B, anteriormost part of spermatozoon: one axoneme, one centriolar derivative, peripheral row of microtubules and ornamentation on membrane. C-G, region of spermatozoon with membrane ornamentation. Microtubules generally as a single row of peripheral parallel units, two axonemes and section of mitochondrion. G shows together a typical section (right) and an intermediary section (left) with ornamentation only on ventral side of spermatozoon. H, longitudinal section in region with membrane ornamentation, showing axoneme with typical trepaxonematan 9 + ”1” structure, mitochondrion with very regular diameter, peripheral microtubules and external ornamentation on cell membrane (microtubules and ornamentation visible on both sides). I-K, various sections at the limit of region with ornamentation and region without ornamentation and additional microtubules. A section of mitochondrion is visible in all sections; a section of nucleus appears, with very small diameter. L-R, sections in the region posterior to membrane ornamentation, showing decreasing number of external microtubules and progressive appearance of section of nucleus. Scale in K, valid for all figures: 500 nm.

**Figure 10:**
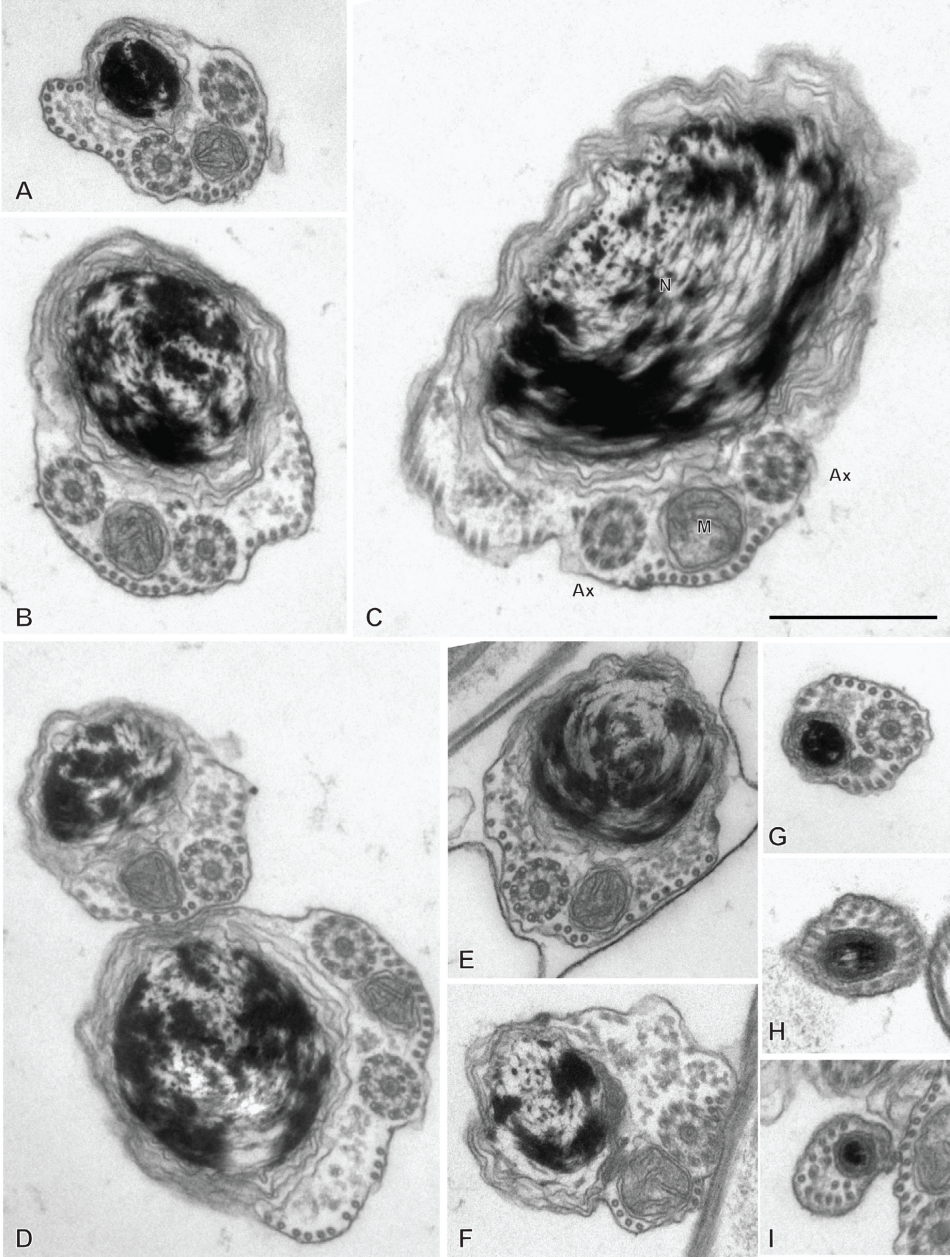
Spermatozoon of *Rajonchocotyle emarginata*, posterior part. A-I, transverse sections, in antero- posterior sequence. A-C, section with two axonemes, mitochondrion, nucleus with various degrees of dilatation, and peripheral microtubules. The nuclear envelope often has additional membranes. D-F, sections with a single axoneme, mitochondrion, nucleus, and peripheral microtubules. G, single axoneme, section of nucleus with dense chromatin and small diameter, mitochondrion with very small diameter, and peripheral microtubules. H, I, extremity with section of nucleus and no mitochondrion. A few microtubules present. Scale in C, valid for all figures: 500 nm.

The mature spermatozoon is a very elongate and thin cell (Figures 9 and 10), with, according to our reconstitution of the anteroposterior sequence of transverse sections, a wider posterior extremity containing the nucleus (Figure 10). The axonemes show a typical trepaxonematan 9 + “1” structure (Figures 9 and 10, transverse sections; Figure 9H, longitudinal section).

The general structure of the spermatozoon is biflagellate, with two axonemes incorporated into the sperm body, a longitudinal mitochondrion, a nucleus, and peripheral longitudinal microtubules. The anterior extremity of the spermatozoon is thinner than the rest; it contains a single axoneme and peripheral microtubules (Figure 9A,B). The anterior region is identical to the elongate late zone of differentiation shown in Figure 8, with a circular section, an almost continuous circle of longitudinal parallel microtubules associated with characteristic external ornamentation on the membrane, two axonemes and a section of the mitochondrial ribbon (Figure 9C-G); longitudinal sections show that the mitochondrion is a regular ribbon and that the external ornamentation looks like a continuous fuzzy layer (Figure 9H). A section of the nucleus with very small diameter, reduced to the nuclear envelope without electron-dense chromatin, is seen in all sections except the anteriormost ones (Figure 9C-Q). More posteriorly, the external ornamentation progressively disappears and the continuous row of peripheral microtubules is gradually replaced by two sets of microtubules without ornamentation, one dorsal and one ventral; attachment zones are visible, thus indicating that this region originates from the fusion of the free flagella with the median cytoplasmic process (Figure 9I, 9L-Q). A part of this region has very few microtubules and sections are more oval than triangular in shape (Figure 9O-Q). The posterior region of the spermatozoon contains the same elements (two axonemes, peripheral microtubules and a section of the ribbon-shaped mitochondrion) but the section of the nucleus represents an increasing portion of the section (Figure 9R, 10A-D). At its wider part, the nucleus has additional membranes around the nuclear envelope and fibrous chromatin. The posterior part of the spermatozoon shows the same structure but only one axoneme is present (Figure 10E-G). The posterior extremity contains a section of the nucleus with small diameter and only a few microtubules (Figure 10H, I).

**Figure 14:**
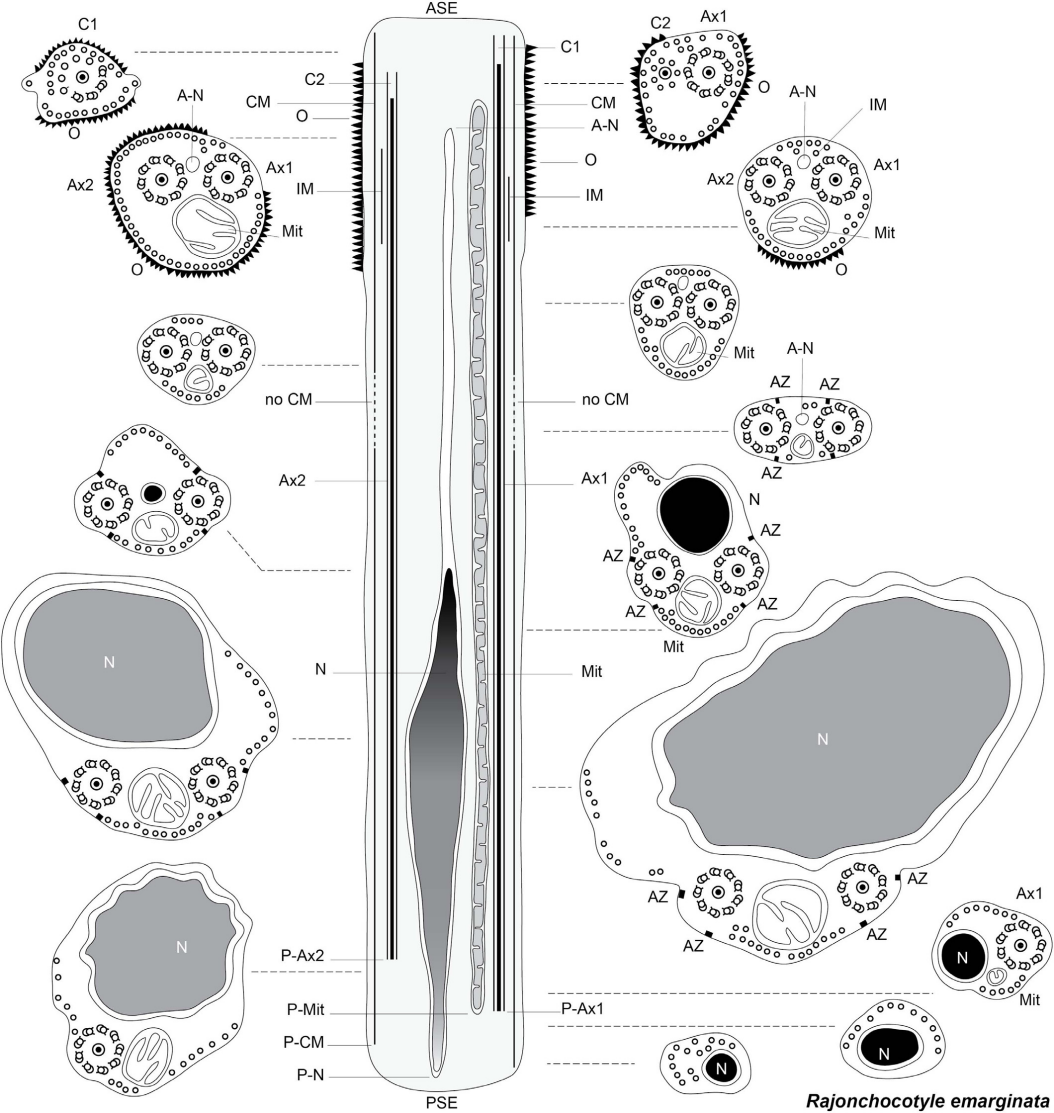
Schematic reconstruction of the spermatozoon of *Rajonchocotyle emarginata.*

Figure 14 is a schematic drawing of our interpretation of the structure of the mature spermatozoon of *Rajonchocotyle emarginata*.

### Spermatozoon of the hexabothriid *Callorhynchocotyle callorhynchi* (Figures 11 and 12)

**Figure 11:**
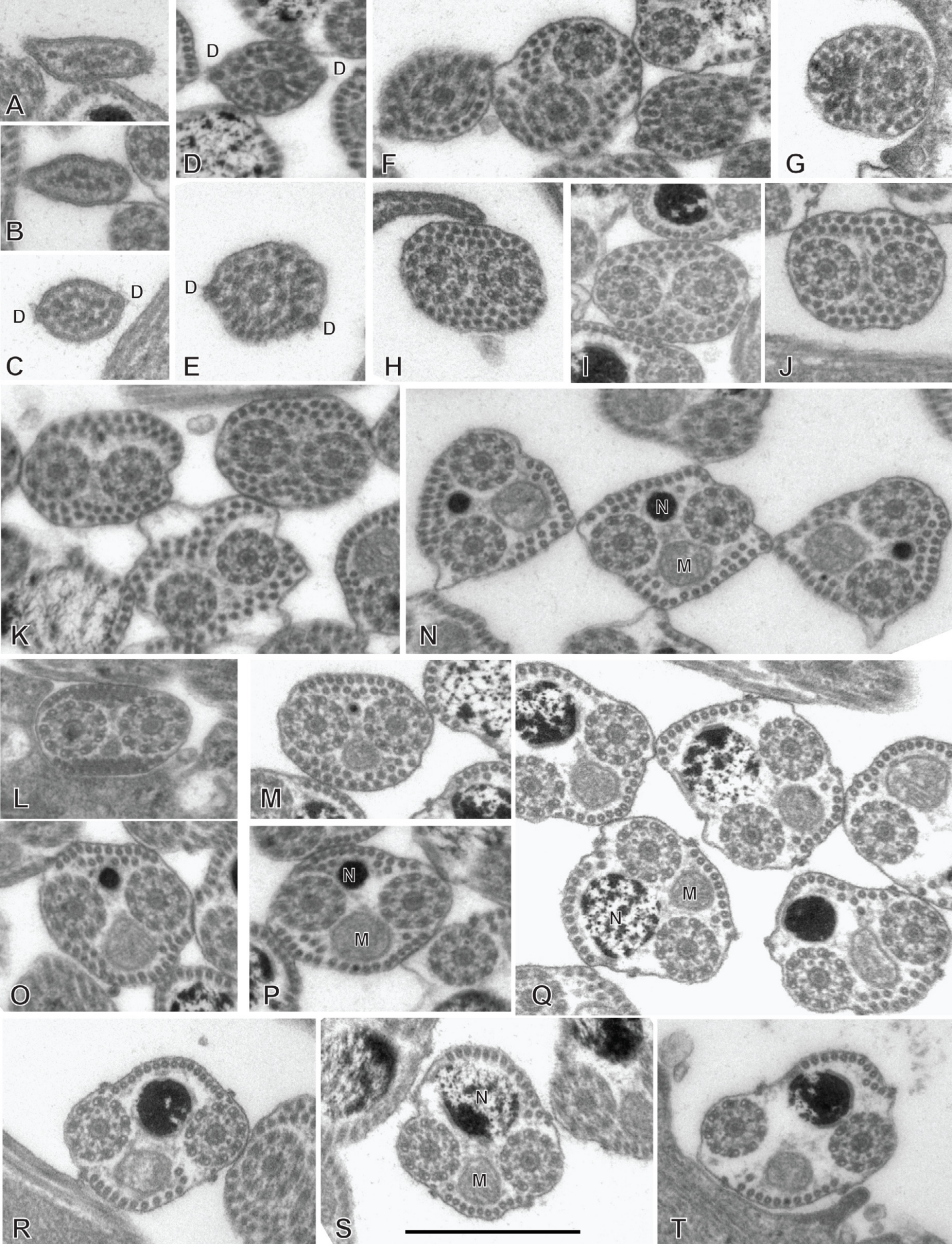
Spermatozoon of *Callorhynchocotyle* *callorhynchi*, anterior part. A-T, transverse sections, in antero-posterior sequence. A-C, anteriormost sections, showing a few microtubules. D, E, centriole and first axoneme. F, two sections with single axoneme surrounding a section with two axonemes. G, one fully formed axoneme and centriole of other axoneme. H-L, sections with two axonemes, peripheral microtubules on dorsal and ventral part, and additional microtubules. M-P, sections with peripheral microtubules on dorsal and ventral part, progressive disappearance of additional microtubules, appearance of sections of mitochondrion and nucleus. Q-T, “typical” sections of spermatozoon, showing two axonemes, one section of mitochondrion, one section of nucleus, ventral and dorsal peripheral microtubules and a few scattered additional microtubules. Scale in S, valid for all figures: 500 nm.

**Figure 12:**
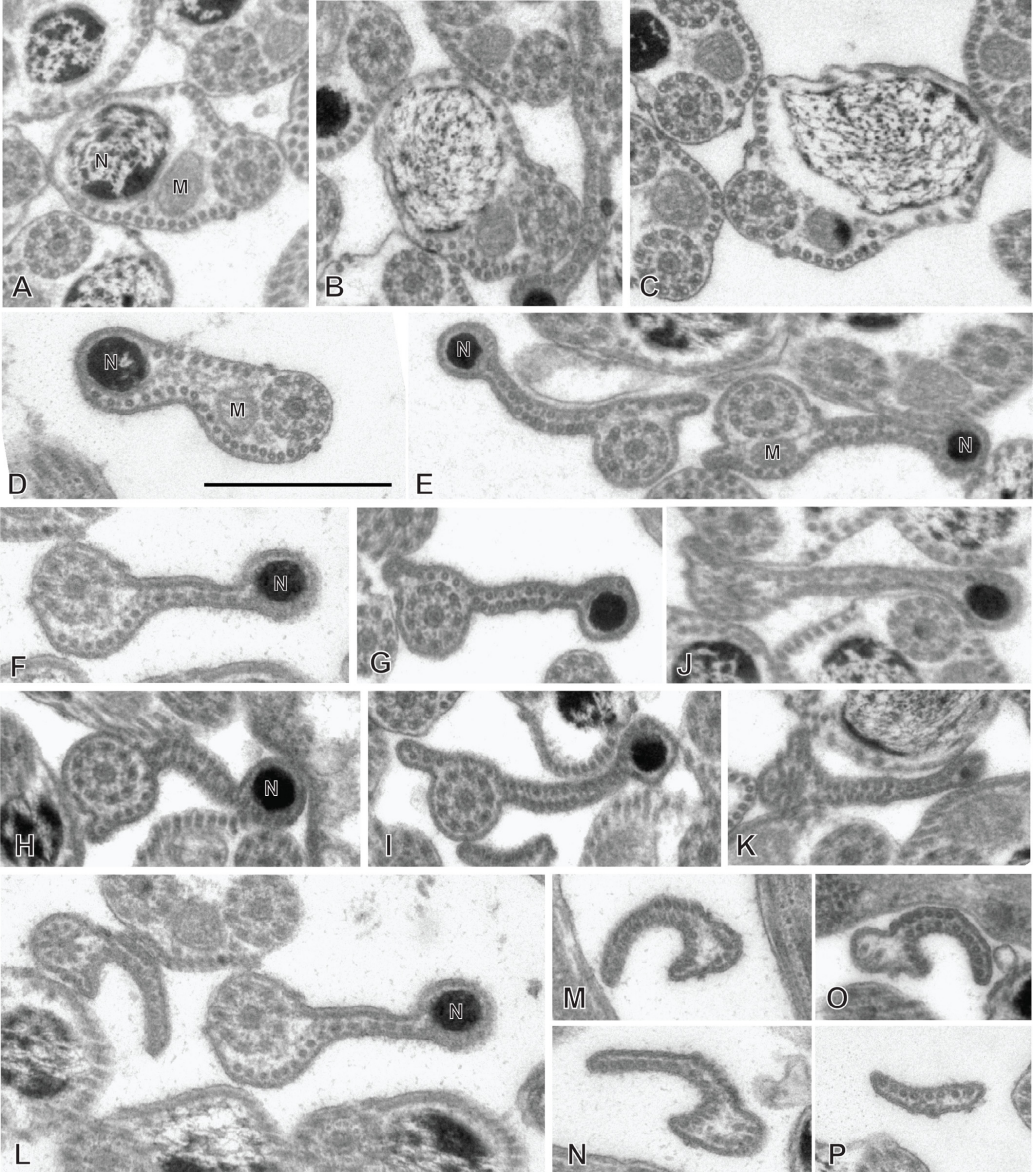
Spermatozoon of *Callorhynchocotyle* *callorhynchi*, posterior part. A-P, transverse sections, in antero-posterior sequence. A-C, similar to “typical” sections of spermatozoon, with one section of mitochondrion, one section of nucleus, ventral and dorsal peripheral microtubules and a few scattered additional microtubules, but with a single axoneme. The nucleus chromatin is not electron-dense. D, intermediary region in which the section is not flattened in the middle; one axoneme, ventral and dorsal peripheral microtubules, section of mitochondrion with very small diameter, and section of nucleus with electron-dense content. E-I, various sections in the flattened region, with single axoneme, ventral and dorsal peripheral microtubules, no mitochondrion, nucleus as small diameter electron-dense section. J-K, flattened region, distal extremity of axoneme; the section of the nucleus has a very small diameter or almost disappeared (K). L, side-by-side section in flattened region and distal comma-shaped section with axoneme reduced to a circle of single microtubules. M-O, distal comma-shaped section, no axoneme. P, distalmost section. Scale in E, valid for all figures: 500 nm.

Spermiogenesis was not observed in this species. The mature spermatozoon is a very elongate and thin cell (Figures 11 and 12), with, according to our reconstitution of the anteroposterior sequence of transverse sections, a larger posterior extremity containing the nucleus and a particular region containing what looks like an undulating membrane. The axonemes show a typical trepaxonematan 9  + “1” structure (Figures 11 and 12).

The general structure of the spermatozoon is biflagellate, with two axonemes incorporated into the sperm body, a longitudinal mitochondrion, a nucleus, and peripheral longitudinal microtubules. The anterior extremity of the spermatozoon is thin; it contains only a few microtubules (Figure 11A-C). More posteriorly, the first axoneme appears (Figure 11D, E) followed by the second axoneme (Figure 11G). At the level of the first centriole and slightly posterior to it, two dense zones are visible within the cytoplasm, outside the microtubule row (Figure 11C-F). The anterior region of the spermatozoon contains the two axonemes and numerous microtubules, organized at two rows of dorsal and ventral microtubules, each doubled by a more internal row of microtubules (Figure 11H-K); no cortical microtubules are present on the sides. This region does not contain a section of the mitochondrion nor of the nucleus. Progressively, this region is transformed in a region with two sets of microtubules, one ventral and one dorsal, devoid of the internal row of microtubules; a section of the mitochondrion and a section of the nucleus appear (Figure 11L-P); intermediary sections (Figure 11L-P) show that the second, internal row of microtubules is progressively lost. The next region has only the “typical” elements of a mature neodermatan spermatozoon, *i.e.* two lateral axonemes, a mitochondrion, a nucleus, and two rows of peripheral microtubules, one on the ventral and one on the dorsal side (Figure 11Q-T). More posteriorly, one of the axonemes disappears and sections contain only a single axoneme, a section of mitochondrion and nucleus, and dorsal and ventral peripheral microtubules; the nucleus has a wide section with fibrous chromatin (Figure 12 A-C). The next region is quite particular: the shape of transverse sections progressively flattens, with the axoneme on one side, the section of the nucleus on the other side, and a thin layer of cytoplasm containing only the two rows of peripheral microtubules, now close one to the other, between the axoneme and the nucleus (Figure 12D-I). Most posterior sections show the axoneme ending as singlets (Figure 12K,L). The posterior extremity shows only a comma-shape section with peripheral microtubules (Figure 12 M-O), then a small structure with only a few microtubules (Figure 12P).

**Figure 15:**
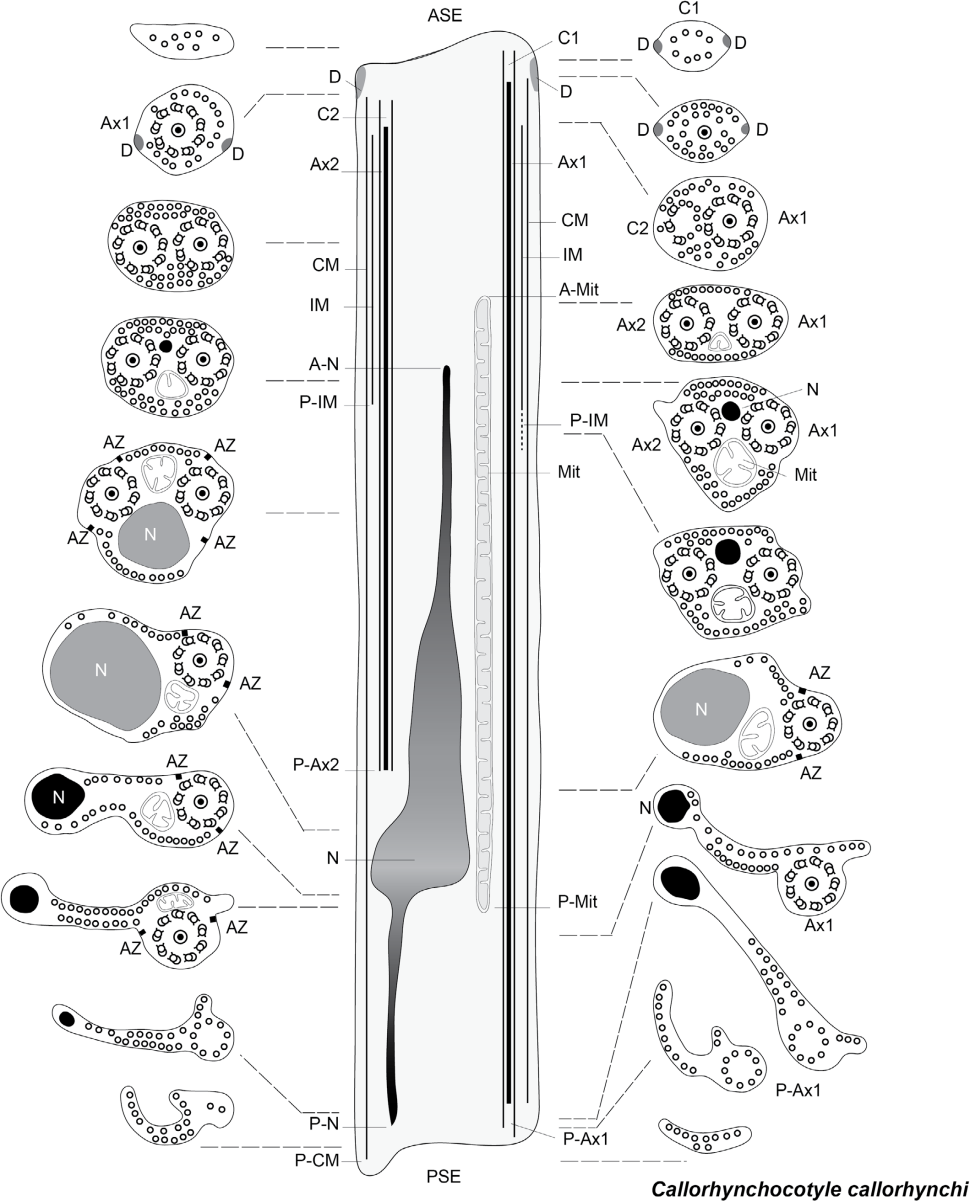
Schematic reconstruction of the spermatozoon of *Callorhynchocotyle* *callorhynchi*.

Figure 15 is a schematic drawing of our interpretation of the structure of the mature spermatozoon of *Callorhynchocotyle callorhynchi*.

## Discussion

### Significance of variations of sperm ultrastructure within the Polyopisthocotylea

The ultrastructure of spermatozoa has been considered as relatively homogeneous among polyopisthocotyleans [35,46], and sperm structure did not provide any character useful for understanding the relationships between the various families of polyopisthocotylean monogeneans [34,42]. This is a contrasting situation to what was found in the monopisthocotyleans, in which the structure is highly variable [16,17,24,39,44,46,49–53,57,59–63,65,66,71,72,76,77,93,98,101–103,106–108] and makes it possible to distinguish synapomorphies for certain families or groups of families [34,35,38,39,42,43,46,57,61,66].

In most polyopisthocotylean monogeneans, the principal region of the spermatozoon (*i.e.* the region with the nucleus and mitochondrion) has two axonemes and a continuous row of peripheral microtubules. A single significant divergence from this pattern was found in the Diplozoidae [47,48]. Diplozoids are unique in the monogeneans (and even in the animal world) in that the two hermaphrodite members of a pair are united for life, with the genital ducts in permanent communication [15]. Sperm morphology is known to be driven by constraints of sperm function and competition [25]; it was hypothesized [39,47] that the aberrant sperm structure in diplozoids was the result of the absence of sperm competition, and thus more representative of a variation of fertilization biology than the mark of a distinctive diplozoid branch in the polyopisthocotylean tree. A parallel situation is also encountered in the schistosomes (Digenea) [31,36,39,45].

A special structure found in rare polyopisthocotyleans is an undulating membrane. A typical undulating membrane was described in *Gotocotyla* sp. [56]. This undulating membrane is composed of a lateral flange containing more than one hundred parallel microtubules, and observations of living spermatozoa showed that the undulating membrane was functional, *i.e.* showing active motility [56]. This was considered an autapomorphy of *Gotocotyla* [34]. Other structures superficially looking like this undulating membrane have been mentioned in various polyopisthocotyleans, generally in the form of a lateral flange with longitudinal peripheral microtubules. Examples, listed by Quilichini *et al.* [89] are *Pricea multae*, *Discocotyle sagittata* and *Concinnocotyla australensis* (references in Table 1); none of these are similar to what exists in *Gotocotyla*, and none have been shown to be functional. A single flange is found in *P. multae* and *D. sagittata*, but there are two flanges in *C. australensis*. We have no evidence that these various lateral flanges are homologous between themselves, and they should probably be considered as autapomorphies of the various taxa in which they were found. The flat sections described here in the spermatozoon of *Callorhynchocotyle callorhynchi* are reminiscent of what is called, in urodele amphibians, an “undulating membrane” [3,23]. This structure is different from all other cases found in monogeneans since it includes the nucleus and a single axoneme, with a flattened part between them with longitudinal microtubules; we have no information whether this structure is functional or not, *i.e.* whether it has a special role in the movement of the spermatozoon. We consider it to be an autapomorphy of *Callorhynchocotyle*, and we point out that, given the current state of knowledge on hexabothriid sperm, it is not a synapomorphy of the Hexabothriidae since it was not found in *Rajonchocotyle emarginata*. We also point out that it does not resemble and is not homologous to the undulating membrane of *Gotocotyla* and the lateral flanges of certain other polyopisthocotyleans.

Various modifications of the nucleus shape, especially in cross sections, have been described. These include a crescent-shaped nucleus partially surrounding the axonemes, in *Discocotyle sagittata*, an annular nucleus completely surrounding the axonemes in *Concinnocotyla australensis*, and a special polygonally shaped nucleus in *Atriaster* spp. (references in Table 1). These variations should be considered autapomorphies of the taxa in which they were found. It is possible that variations of the structure of the anterior and posterior extremities of the spermatozoon provide additional structures useful for phylogenies [89], but this information is often lacking in published papers.

### Significance of variations of sperm ultrastructure within the Neodermata and the Polyopisthocotylea

The Neodermata, which include all major groups of parasitic Platyhelminthes, *i.e.* the Digenea, Aspidogastrea, Eucestoda, Gyrocotylidea, Amphilinidea, Polyopisthocotylea, and Monopisthocotylea (the latter two often considered as forming the Monogenea) are characterised by spermatozoal synapomorphy, the proximo-distal fusion of axonemes during spermiogenesis [35]. Associated with this process is the presence of a characteristic structure termed the zone of differentiation [35,39]. The spermatozoon of the Neodermata has typically two axonemes and longitudinal peripheral microtubules [35,39].

**Figure 16:**
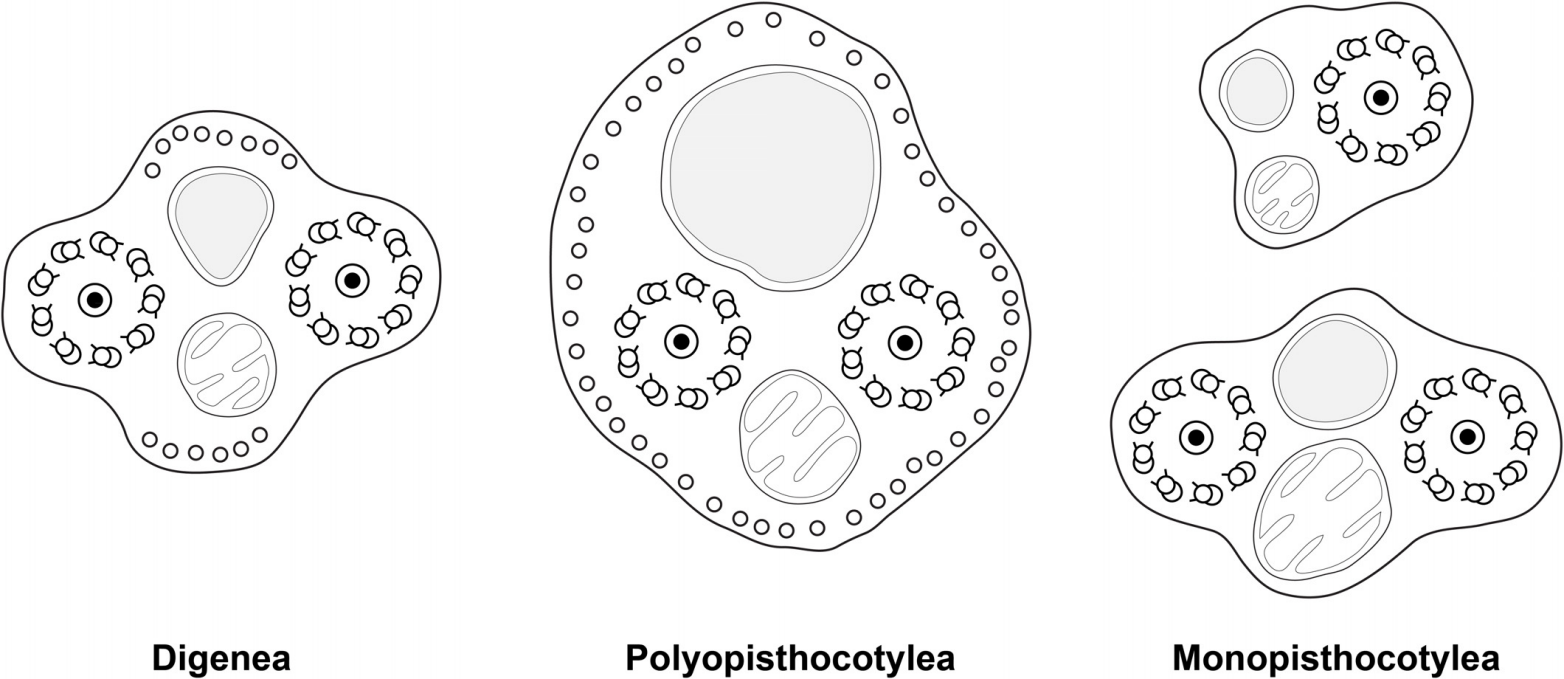
Diagrams of spermatozoa (redrawn from Justine, 1991 [35]). Caption adapted from original caption of figure. Diagrams were drawn from original micrographs of transverse sections. Digenea. Dorsal and ventral microtubules are present (proposed as a synapomorphy for the Cercomeridea). There are no lateral microtubules (symplesiomorphic compared with the synapomorphy for the polyopisthocotylean Monogenea). Polyopisthocotylea. Dorsal and ventral microtubules are present (synapomorphy for Cercomeridea). Note the presence of lateral microtubules (proposed as a synapomorphy for the Polyopisthocotylea). Monopisthocotylea (uniflagellate and biflagellate). Microtubules are absent from the principal region of the spermatozoon, which is interpreted as (i) the absence of dorsal and ventral microtubules, a reversal of the synapomorphy for the Cercomeridea; and (ii) the absence of lateral microtubules, the symplesiomorphic state versus the synapomorphy for the Polyopisthocotylea.

Within the Neodermata, Justine (1991) [35] considered that there was a plesiomorphic pattern, with two axonemes and ventral and dorsal microtubules in the “principal region” of the spermatozoon, *i.e.* the region which contains the nucleus; it should be kept in mind that since the sperm of the Neodermata is “inverted”, the region with the nucleus is posterior [35,39]. This plesiomorphic pattern is found in the Digenea and Cestoda. Two synapomorphies, defining major groups, were proposed: absence of the dorsal and ventral microtubules for the Monopisthocotylea, and presence of additional lateral microtubules for the Polyopisthocotylea. Figure 16 was drawn from Figure 5 in the 1991 paper by Justine [35].

For the Polyopisthocotylea, the synapomorphy “presence of lateral microtubules in the principal region of the spermatozoon” was defined by Justine in 1991 [34,35,38,39]. It was used in the two major attempts to develop a phylogeny of the monogeneans by Boeger & Kritsky in 1993 [12] and 2001 [14]; we will discuss mainly the more recent of these papers [14] because it includes the matrix. According to Boeger & Kritsky [14], this sperm synapomorphy was one of the six synapomorphies uniting the Polyopisthocotylea. It was used as character number 64 in their analysis as “lateral microtubules in the spermatozoon principal region of” and was character change 132 in their hypothesis [14]. Apomorphic state “present” was considered one of the six character changes that separates the Heteronchoinea (= Polyopisthocotylea) from the rest of the monogenes, considered as Polyonchoinea (= Monopisthocotylea); for equivalences between Polyopisthocotylea and Monopisthocotylea and between Heteronchoinea and Polyonchoinea see Table 1 in [75].

The present findings introduce an important change in the character matrix since this character was not found in the present study in the Chimaericolidae and Hexabothriidae, two of the most basal groups in the Polyopisthocotylea. Our knowledge of sperm structure in hexabothriids was limited to *Erpocotyle catenulata*; however, the published observation on this species by Tuzet & Ktari in 1971 [105] is a single micrograph, somewhat fuzzy, of spermatozoa which are clearly altered with open membranes; peripheral microtubules are present, but the presence of lateral microtubules in sections with a nucleus cannot be ascertained. We thus conclude that the character “lateral microtubules in principal region of spermatozoon” is not present in the hexabothriid species studied so far, which belong to three genera, *Erpocotyle*, *Callorhynchocotyle* and *Rajonchocotyle*.

Moreover, careful re-analysis of accounts of sperm structure in the Polystomatidae shows that this character was not general in this family. It can be found in *Concinnocotyla australensis*, but cannot be seen in any of the photographs of spermatozoa of *Pseudodiplorchis americanus* or *Polystoma* sp.; in *Polystoma spratti*, the microtubules at the level of the nucleus never form a complete row (references in Table 1). For the Sphyranuridae, our knowledge is limited to observations without illustrations [34]. Interpretation of the significance of presence and absence of the character in the Polystomatoinea would need additional observations; we provisionally consider that presence of the row of microtubules in certain polystome species has its origin in independent evolutionary events or convergences.

It thus appears that the apomorphic character “presence of lateral microtubules in the principal region of the spermatozoon” is not a synapomorphy of the Polyopisthocotylea as a whole.

### Discrepancies between actual data and the matrix of Boeger & Kritsky (2001)

A close examination of the character matrix in Boeger & Kritsky [14] shows that the character state was, in many cases, not coded in accordance with the data available at the time of publication of this paper (2001). The matrix includes 57 lines; for the Polyopisthocotylea, 33 families are listed, and the character state was coded as “1” (presence of lateral microtubules) in 32 of them − the only exception was the Montschadskyellidae, coded as “?”, which was and is still correct. A comparison with Table 1 shows that the coding was correct in 15 families (*i.e.* character state “1” was actually visible in published papers), but that 13 families were erroneously coded “1” whereas the character state was unknown. Moreover, for two families, Pterinotrematidae and Axinidae, the character was coded as “1” whereas published information was 0. We interpret these discrepancies as “over-generalization”, *i.e.* a character state common in a group was coded for all members of the group, while it was in fact present only for some members. In the matrix by Boeger & Kritsky [14], over-generalization concerns 15 polyopisthocotylean families amongst 33 (Table 2). The inspiration for the over-generalization of spermatological characters can probably be found in the papers on sperm structure by Justine [34,35,39,46], who generally considered that sperm structure was homogeneous in the Polyopisthocotylea. Over-generalization and errors in the analysis of sperm characters probably calls for a re-examination of some other (non-spermatological) characters in the matrix of Boeger & Kritsky [14]. However, it should be outlined that small corrections in spermatological characters in the matrix would probably not change the resulting tree and the phylogeny proposed by Boeger & Kritsky [14].

**Table 2:** State of character “presence of lateral microtubules in principal region of spermatozoon” in families of the Polyopisthocotylea. A comparison between the state used in Boeger & Kritsky (2001)’s phylogeny [14] and the actual information which was available at that time (see Table 1) and current information after present paper.

Family	State in Boeger & Kritsky, 2001, Appendix 10.2	Actual information (according to Table 1)	Comments	Current information (according to present paper)
Polystomatidae	1	0 or 1	over-generalization	
Sphyranuridae	1	dubious	over-generalization	
Chimaericolidae	1	unknown	over-generalization	state = 0
Diclybothriidae	1	unknown	over-generalization	
Hexabothriidae	1	dubious	over-generalization	state = 0
Pterinotrematidae	1	0	error	
Mazocraeidae	1	1	confirmed	
Hexostomatidae	1	1	confirmed	
Plectanocotylidae	1	1	confirmed	
Mazoplectidae	1	unknown	over-generalization	
Discocotylidae	1	1	confirmed	
Diplozoidae	1	1	homology uncertain − different sperm structure	
Diclidophoridae	1	1	confirmed	
Anthocotylidae	1	unknown	over-generalization	
Gastrocotylidae	1	1	confirmed	
Chauhaneidae	1	1	confirmed	
Protomicrocotylidae	1	1	confirmed	
Gotocotylidae	1	1	confirmed	
Microcotylidae	1	1	several genera and species − confirmed in all	
Heteraxinidae	1	1	confirmed	
Allopyragraphoridae	1	unknown	over-generalization	
Diplasiocotylidae	1	unknown	over-generalization	
Axinidae	1	0	error	
Pyragraphoridae	1	1	confirmed	
Montschadskyellidae	?	unknown	absence of information confirmed	
Pseudodiclidophoridae	1	unknown	over-generalization	
Neothoracotylidae	1	unknown	over-generalization	
Bychowskycotylidae	1	unknown	over-generalization	
Allodiscocotylidae	1	unknown	over-generalization	
Rhinecotylidae	1	unknown	over-generalization	
Heteromicrocotylidae	1	unknown	over-generalization	
Octomacridae	1	1	confirmed	
Pseudomazocraeidae	1	1	confirmed	

### Re-interpretation of the major synapomorphies of the Monogenea in light of this study

Hypotheses for the phylogeny of the Neodermata, the parasitic Platyhelminthes which include the Cestoda, Trematoda and Monogenea, have been based, successively or simultaneously, on four sets of characters over the years: (1) Traditional morphology; (2) Sperm ultrastructure; (3) Sequences of selected parts of the genome; (4) Complete mitochondrial genomes. We can expect that the next step will be the comparison of complete genomes; however, we believe that main morphological or spermatological characters are still to be considered even in the future.

Major phylogenies based on morphology by Boeger & Kritsky (with the inclusion of several characters of spermatozoa) have concluded on monophyly of the Monogenea [12,14]. However, spermatological characters do not provide evidence for monophyly [35,39,40,42,43,46] and a reexamination of morphological characters found the monogenean non-monophyletic [22]. Molecular analyses based on 18S or 28S partial gene sequences [74,75,79] found the Monopisthocotylea and the Polyopisthocotylea each to be monophyletic, but the Monogenea were paraphyletic; an exception is the analysis by Lockyer *et al.* (2003) [69] in which the Monogenea were monophyletic. Complete mitochondrial genomes are known for about 40 species of Platyhelminthes [100], including a dozen monogeneans [4,30,64,80–82,111–114]. Ye *et al.* (2014) [111] compared the mitogenomes of 10 species and found that Polyopisthocotylea and Monopisthocotylea had distinct gene arrangements. Therefore, molecular data currently available still point towards non-monophyly of the Monogenea. It is important to mention that all mitogenomes available for the Polyopisthocotylea are from a single superfamily, the Mazocraeidea, a situation which is reminiscent of the database on sperm ultrastructure before the present study.

**Figure 17:**
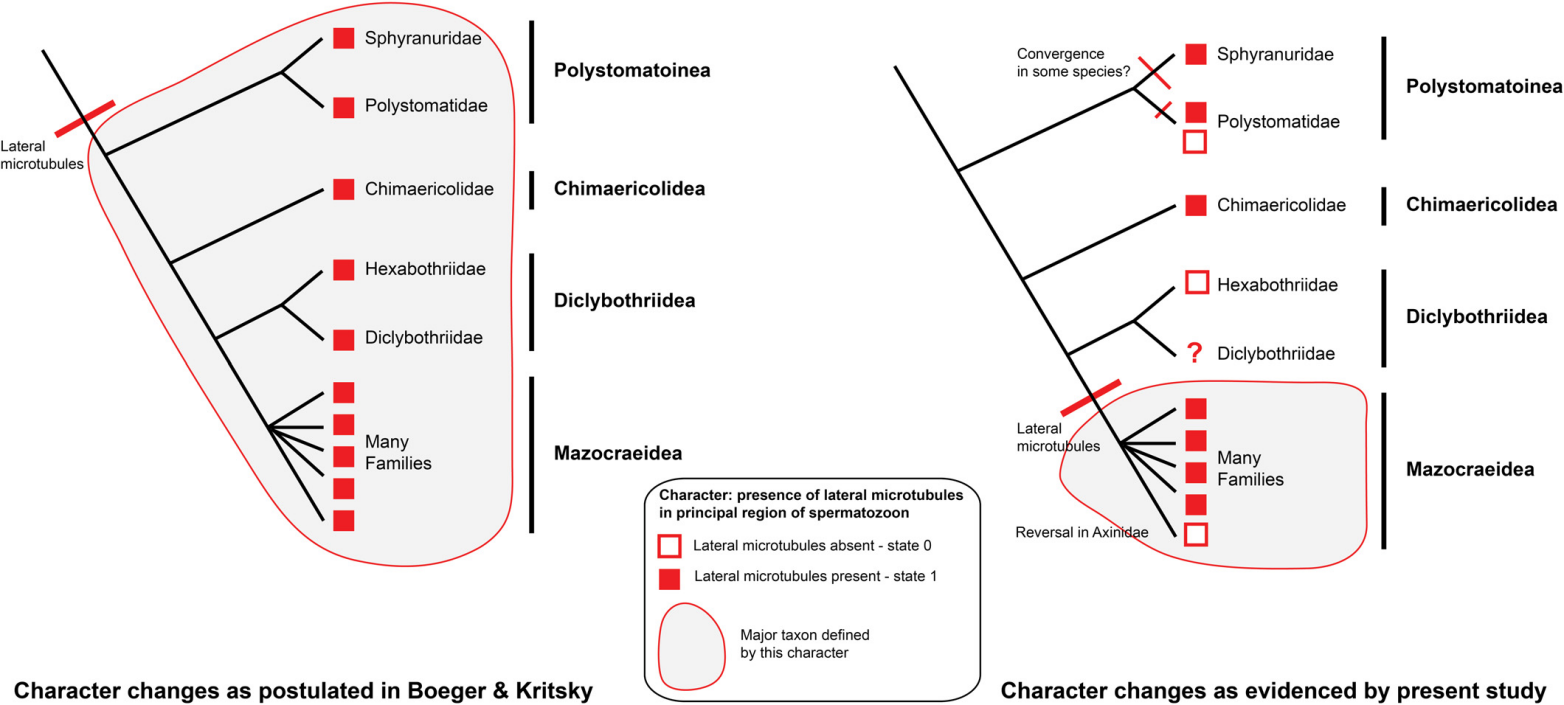
Interpretation of synapomorphies of spermatozoa, placed on a phylogeny of the Polyopisthocotylea. Left: Character changes as postulated in Boeger & Kritsky (2001) [14]. The taxon defined by the character “presence of lateral microtubules in principal region of spermatozoon” is the Polyopisthocotylea as a whole. Right: Character changes as evidenced by present study. The taxon defined by the character “presence of lateral microtubules in principal region of spermatozoon” is restricted to the Mazocraeidea.

In Figure 17, we try to reconcile the present information with a general phylogeny of the Polyopisthocotylea. We consider, finally, that the apomorphic character “presence of lateral microtubules in the principal region of the spermatozoon” is a synapomorphy of the Mazocraeidea. An investigation of the Diclybothriidae, the sister-group of the Hexabothriidae would be of interest; investigations in additional hexabothriids would be useful too, since our present knowledge is based only on three genera among the fifteen included in the family.

We believe that the present findings and interpretation are important not only for the polyopisthocotylean monogeneans, but for our understanding of phylogeny in the whole Neodermata.

## Conflict of interest

The Editor-in-Chief of Parasite is one of the authors of this manuscript. COPE (Committee on Publication Ethics, http://publicationethics.org), to which Parasite adheres, advises special treatment in these cases. In this case, the peer-review process was handled by an Invited Editor, Jerôme Depaquit.

**Abbreviations for all figures**
A-CM: Anterior extremity of cortical microtubules
A-IM: Anterior extremity of internal microtubules
AM: Arching membrane
A-Mit: Anterior extremity of mitochondrion
A-N: Anterior extremity of nucleus
ASE: Anterior spermatozoon extremity
Ax, Ax1, Ax2: Axonemes
AZ: Attachment zone
C, C1, C2: Centrioles
CM: Cortical microtubules
D: Dense zone
FF: Free flagellum
IB: Intercentriolar body
IM: Internal microtubules
M: (in micrographs), Mit (in diagrams) Mitochondrion
MCE: Median cytoplasmic process
MT: microtubules
N: Nucleus
O: Ornamentation on membrane
P-Ax1, P-Ax2: Posterior extremity of axoneme 1, 2
PSE: Posterior spermatozoon extremity
P-CM: Posterior extremity of cortical microtubules
P-IM: Posterior extremity of internal microtubules
P-Mit: Posterior extremity of mitochondrion
P-N: Posterior extremity of nucleus
SR: Striated root
